# Beyond conventional indicators: Reproductive coercion and its overlap with measured intimate partner violence in Bangladesh

**DOI:** 10.1371/journal.pone.0360372

**Published:** 2026-10-21

**Authors:** Md. Nuruzzaman Khan

**Affiliations:** 1 Department of Population Science, Jatiya Kabi Kazi Nazrul Islam University, Bangladesh; 2 Nossal Institute for Global Health, Melbourne School of Population and Global Health, The University of Melbourne, Australia; Independent Global Health Consultant, INDIA

## Abstract

**Background:**

Reproductive coercion is an under-recognised form of violence against women and is not routinely captured by standard measures of intimate partner violence, which commonly focus on physical, sexual, and emotional violence. This study examined the association between reproductive coercion and these measured forms of intimate partner violence and estimated the proportion of women who reported reproductive coercion without reporting any of the physical, sexual, or emotional violence items included in the survey.

**Methods:**

We analysed nationally representative data from 24,951 ever-married women, including 22,161 currently married women, in the Bangladesh Violence Against Women Survey 2024. Outcomes were physical, sexual, emotional, and any conventional IPV, assessed in the past 12 months among currently married women and over the lifetime among ever-married women. Exposures were reproductive coercion pattern and contraceptive coercion typology. Survey-weighted Poisson regression with robust standard errors estimated adjusted prevalence ratios (aPRs) with 95% CIs for associations with conventional IPV. Survey-weighted logistic regression examined factors associated with reproductive coercion without reported conventional IPV and was considered exploratory because of the small number of events. Survey-weighted prevalence estimates were applied to 2022 census population counts to derive population estimates with 95% uncertainty intervals (UIs).

**Findings:**

In the past 12 months, 27.30% of currently married women reported conventional IPV and 3.59% reported reproductive coercion; corresponding lifetime prevalences among ever-married women were 60.40% and 9.25%. Including reproductive coercion increased the prevalence of any reported abuse to 28.61% and 61.18%, respectively. Reproductive coercion was associated with a higher prevalence of each form of conventional IPV, with the largest aPRs among women reporting multiple forms of reproductive coercion. Reproductive coercion without reported conventional IPV was reported by 1.31% (95% CI 1.08–1.59) of currently married women in the past 12 months and 0.78% (0.60–1.01) of ever-married women over the lifetime, corresponding to approximately 645,483 (95% UI 532,154–783,449) and 437,414 (337,106–567,462) women, respectively.

**Interpretation:**

Reproductive coercion captures a dimension of violence against women that is only partly reflected in conventional IPV measures. Women reporting reproductive coercion without conventional IPV might have experienced unmeasured or unreported violence; therefore, this group indicates a measurement gap rather than absence of other abuse. Including validated reproductive coercion measures in national violence surveys could improve evidence for health, legal, and social support responses.

## Introduction

Violence against women (VAW) remains one of the most widespread violations of human rights and a major public health challenge globally. It encompasses a broad range of gender-based acts that cause physical, sexual, psychological, or reproductive harm and is increasingly recognised as arising from unequal gender relations and broader social, cultural, economic, and legal structures that constrain women's autonomy [1]. The World Health Organization estimates that 27% of ever-partnered women aged 15–49 years worldwide have experienced physical and/or sexual intimate partner violence (IPV) during their lifetime, while 13% experienced such violence in the previous 12 months [1]. The burden is disproportionately concentrated in low- and middle-income countries (LMICs), where structural gender inequality, limited legal protection, economic dependence, and weak support systems often intensify women’s vulnerability [2]. In the South Asia region, pooled estimates suggest that approximately one in three ever-partnered women have experienced lifetime IPV, with substantial heterogeneity across countries [1,2]. National evidence indicates persistently high levels of IPV in Bangladesh. The 2015 Bangladesh Violence Against Women Survey reported that 72.6% of ever-married women had experienced at least one form of violence by an intimate partner during their lifetime and 54.7% in the previous year, although estimates varied by violence type and measurement domain [3]. More recent population data continue to show that emotional, physical, and sexual violence remain common among Bangladeshi women, indicating that VAW is an enduring national priority [4].

Although the magnitude of VAW is well established, estimates depend heavily on what forms of violence are measured. Most LMICs rely on standardized modules derived from the World Health Organization and the Demographic and Health Surveys (DHS) Programme, which primarily capture physical, sexual, and emotional partner violence, with some surveys also measuring controlling behaviours [1,5,6]. These standardised instruments have greatly improved global surveillance and comparability across settings but were not designed to comprehensively capture reproductive coercion as a distinct form of partner violence. Reproductive coercion refers to behaviours that interfere with a woman's reproductive autonomy, including contraceptive sabotage, pregnancy pressure, coerced continuation of pregnancy, forced abortion, and threats related to childbearing decisions [7,8]. Consequently, women experiencing reproductive coercion may be classified as not having experienced partner violence if they do not also report the physical, sexual, or emotional IPV acts measured in these surveys.

Over the past decade, reproductive coercion has become well documented in high-income settings, where prevalence estimates commonly range from 8% to 38% depending on population and timeframe, and where strong associations with unintended pregnancy, contraceptive non-use, poor mental health, and co-occurring IPV have been demonstrated [9–11]. In contrast, evidence from LMICs remains limited because reproductive coercion is rarely included in national violence surveys, including most DHS and dedicated VAW surveys. This omission has important implications. Conceptually, reproductive autonomy is a fundamental component of bodily integrity and sexual and reproductive health and rights [12]. Empirically, excluding reproductive coercion may underestimate survey-based prevalence of partner violence, because women who report reproductive coercion but none of the other measured acts would be classified as unexposed. Consequently, prevalence estimates based only on standard IPV items may not reflect an important dimension of gender-based violence relevant to violence prevention, reproductive health, legal protection, and social policy.

This study is informed by the coercive control framework, which conceptualises intimate partner violence as a sustained pattern of behaviours intended to dominate and restrict a woman's autonomy across multiple domains of life rather than as isolated acts of abuse [13]. Within this framework, reproductive coercion represents one manifestation of coercive control alongside physical, sexual, and emotional violence [14,15]. These behaviours frequently co-occur because they arise from the same underlying dynamics of power and control, but they do not necessarily occur together. Women may therefore experience reproductive coercion in the absence of physical, sexual, or emotional violence measured in conventional surveillance systems [14]. Measuring reproductive coercion independently is therefore essential to capture its distinct contribution to partner-perpetrated coercive control and women's overall experiences of violence, consistent with international frameworks recognising reproductive autonomy as a core component of sexual and reproductive health and rights [16].

Research on VAW in Bangladesh and other LMICs has primarily focused on physical, sexual, and emotional IPV, using cross-sectional household surveys, DHS modules, or WHO-derived instruments [1,2,17,18]. These studies have established strong associations between violence and adverse outcomes including depression, suicidal ideation, maternal morbidity, unintended pregnancy, and reduced healthcare use [17,19–21]. A smaller emerging literature has examined reproductive coercion in humanitarian settings in LMICs, suggesting that coercion related to contraception and pregnancy is not rare and frequently co-occurs with physical, sexual, and emotional violence, supporting the view that reproductive coercion forms part of a broader pattern of partner-perpetrated coercive control [11]. However, this evidence remains fragmented, concentrated in local or high-risk samples, and rarely integrated into national violence surveys. To my knowledge, no nationally representative study from Bangladesh has examined how prevalence estimates change when reproductive coercion is added to standard IPV measures, or whether reproductive coercion is largely reported alongside physical, sexual, and emotional IPV or is also reported by women who do not report these measured forms of IPV. Drawing on the coercive control and reproductive autonomy literature, I therefore hypothesised that: (i) reproductive coercion and conventional IPV would show strong positive co-occurrence, as predicted by the coercive control framework, which conceptualises abusive behaviours as interconnected manifestations of partner control operating across multiple domains of intimate relationships rather than as independent forms of violence; and (ii) a measurable subgroup of women would report reproductive coercion without reporting any of the physical, sexual, or emotional IPV items included in the survey, indicating that reproductive coercion is not fully captured by standard IPV measures. To test these hypotheses, I examined the associations between reproductive coercion and conventional IPV and estimated the proportion and number of women who reported reproductive coercion without reporting any measured conventional IPV.

## Methods

### Study setting and design

This study analysed data from the Violence Against Women Survey 2024 [4], the third nationally representative round of Bangladesh’s dedicated survey series on violence against women, following earlier rounds conducted in 2011 and 2015. The survey was implemented by the Bangladesh Bureau of Statistics with technical support from the United Nations Population Fund (UNFPA). The survey was designed to generate updated evidence on the prevalence, forms, patterns, and correlates of VAW in Bangladesh. It followed internationally recognised methodological standards, drawing on the World Health Organization multi-country study approach and the United Nations guidelines for producing official statistics on VAW.

This nationally representative cross-sectional household survey included women aged 15 years and older to produce reliable estimates at national and divisional levels, as well as by place of residence. Bangladesh is administratively divided into eight divisions that differ in their demographic composition, socioeconomic development, levels of urbanisation, gender norms, and access to health and social services. To capture this, Bangladesh’s eight administrative divisions were subdivided into rural areas, urban areas excluding city corporation areas, and city corporation areas in the survey, creating 28 survey domains for independent estimation. A stratified two-stage cluster sampling design was used. In the first stage, primary sampling units (PSUs) were selected within each domain using probability proportional to size (PSU) methods based on the 2022 Population and Housing Census. In the second stage, households were systematically selected within each sampled PSU.

Within sampled households, a household listing identified eligible women aged 15 years or older who had resided in the household for at least six months. Where more than one eligible woman was present, one woman was randomly selected using a built-in computer-assisted personal interviewing (CAPI) randomisation system. Selected respondents who were unavailable or declined participation were not replaced, preserving the integrity of the sampling design.

Data were collected between March and June 2024 through face-to-face computer-assisted personal interviews (CAPI) conducted by 120 trained female enumerators working in 24 field teams, each led by an experienced supervisor. Enumerators completed a 21-day training programme covering concepts of gender and violence, myths about violence, ethical and safety procedures for respondents and interviewers, interviewing techniques, and the questionnaire and CAPI application, followed by a structured field pre-test. All questions were interviewer-administered, so respondents did not need to read the questionnaire. Interviews were conducted in Bangla. Consistent with WHO and United Nations ethical and safety recommendations for violence research, the survey was introduced at community and household levels under the neutral title “Survey of Women's Status,” the word “violence” was not used in survey materials, only one woman per household was interviewed, and enumerators were assigned outside their home districts. Interviews were conducted only in private settings and were rescheduled if privacy could not be guaranteed. If a husband or another household member entered during an interview, enumerators switched to a decoy women's health questionnaire, a procedure explained to respondents in advance. Verbal informed consent was obtained using a standardised script. Respondents were told that they could skip any question or stop at any time, explicit permission was sought again before the violence modules, and all respondents received information on available support and referral services at the end of the interview. Data quality was monitored through supervisor review, daily central checks, geo-location tracking, and weekly consistency checks. Data from 355 PSUs affected by inconsistencies were re-collected from newly selected households in December 2024 and January 2025. Overall, the survey included approximately 1,440 PSUs and 28,800 households, and 27,476 women completed interviews, yielding a response rate of 95.4%. The broad description of the survey has been published in the survey report [4].

### Analytical sample

Analyses were restricted to ever-married women. This restriction was made in line with the aim of this study to examine association between reproductive coercion and standard measure of IPV as the questions related to reproductive coercion were asked married women only. For analyses of past 12 months experience, the sample was further limited to currently married women, while lifetime analyses included all ever-married women. Women who had never married were excluded. The final analytical sample comprised 24,951 ever-married women for lifetime analyses and 22,161 currently married women for past 12 months analyses. All participants included in the analysis had complete data for all the variables considered.

### Outcome variables

The primary outcomes were conventional measures of IPV, including physical, sexual, and emotional violence. The survey collected data on each domain of IPV using behaviourally specific questions that have been published in the official survey report [4]. Physical violence included acts such as slapping, pushing, hitting, or other forms of physical harm. Sexual violence captured forced sexual intercourse or other unwanted sexual acts. Emotional violence included experiences such as insults, humiliation, intimidation, or threats. For each act, respondents reported whether they had ever experienced it (lifetime, since marriage) and whether it had occurred within the 12 months preceding the survey. As the analytic sample was restricted to ever-married women, all of these items referred to acts by the husband within marriage. Binary indicators were then constructed separately for each domain and recall period, coded as “1” if a woman reported experiencing at least one act within that domain and period, and “0” otherwise. Following this, composite indicators of any conventional IPV measures were generated for both lifetime and past 12 months. These composite variables were coded as “1” if a woman reported at least one act of physical, sexual, or emotional violence, and “0” if none were reported. This approach is consistent with standard practice in violence research and aligns with SDG monitoring frameworks, allowing the identification of women exposed to any form of conventional IPV measure [4].

### Exposure variable

Reproductive coercion was the main exposure variable of interest. The survey collected data on reproductive coercion using items that captured pressure or prevention related to contraception, pregnancy, and abortion, which have also been published in the survey report [4]. Each of these items is consistent with the structure and behaviourally specific items used in reproductive coercion research [8,15].

Separate questions were asked for each behaviour, with yes/no response options, and referred to acts by the husband. For each item, women were asked whether it had ever occurred in their marriage (lifetime) and whether it had occurred in the 12 months preceding the survey; the exact item wording, response options, and recall periods. The reproductive coercion items formed part of the national questionnaire, which was adapted from the United Nations Economic Commission for Europe violence against women module recommended in United Nations Statistics Division guidelines, with selected WHO multi-country study items, through consultations with government, United Nations, and civil society stakeholders and three rounds of pre-testing in urban, rural, and city corporation areas that assessed question wording, comprehension, completion time, and response variation [4]. To my knowledge, the items were field tested for accuracy before conducting the final survey, although no separate cognitive testing or psychometric validation has been performed. Contraceptive coercion included being forced to use contraception against a woman’s wishes, being prevented from using contraception when desired, or experiencing both forms. Pregnancy-related coercion captured pressure or interference related to becoming pregnant, continuing a pregnancy, or being forced or prevented from having an abortion. These items were used to construct measures of reproductive coercion for both the past 12 months and lifetime periods. Binary indicators were first created for each component and recall period, coded as “1” if a woman reported experiencing at least one act within that category and period, and “0” otherwise. Based on these indicators, a composite pattern variable of reproductive coercion was generated with four categories: no reproductive coercion, contraceptive coercion only, pregnancy outcome coercion only, and both contraceptive and pregnancy outcome coercion. A second exposure variable further classified contraceptive coercion into four categories: no coercion, forced contraceptive use only, prevented contraceptive use only, and both forced and prevented contraceptive use. These categories were constructed separately for past 12-month and lifetime recall periods.

### Covariates

The covariates included in this study were selected in two stages. First, I reviewed the relevant literature to identify variables based on prior evidence and their conceptual relevance to violence and reproductive autonomy [17–21]. Second, the availability of these variables in the dataset was assessed. Consistent with the coercive control and gender inequality frameworks, these characteristics were considered potential confounders because they may be related both to reproductive coercion and to exposure to, or reporting of, other forms of IPV. All covariates identified in these two stages were retained in every model irrespective of statistical significance. The final set of covariates comprised individual, partner, household, and geographic characteristics. Individual-level characteristics included respondent age (15–19, 20–29, 30–39, ≥ 40 years), child marriage (marriage before age 18: yes/no), number of living children (none, 1–2, 3–4, ≥ 5), educational attainment (primary or less, secondary, higher secondary or above), employment status (yes/no), religion (Muslim, non-Muslim), and disability status (none, some difficulty, moderate/severe difficulty). Women's employment status was defined as whether the respondent reported currently earning money from work, including informal or home-based income-generating activity, in the 12 months preceding the survey. Disability status was assessed using functional-difficulty categories consistent with the Washington Group Short Set of Questions (difficulty with seeing, hearing, walking, cognition, self-care, and communication), grouped into none, “some difficulty” and “moderate/severe difficulty.” Partner-related variables included husband’s education (primary or less, secondary, higher secondary or above) and husband’s extra-marital relationship (yes/no). Household-level characteristics included household type (non-slum, slum) and wealth quintile (poorest, poorer, middle, richer, richest). Geographic variables included place of residence (rural, urban) and administrative division (Barishal, Chattogram, Dhaka, Khulna, Mymensingh, Rajshahi, Rangpur, Sylhet).

### Statistical analysis

All analyses accounted for the complex survey design by incorporating sampling weights, clustering, and stratification. Weighted proportions with 95% confidence intervals (CIs) were used to describe sample characteristics and estimate the prevalence of conventional IPV, reproductive coercion, and reproductive coercion without reported conventional IPV for both past 12-month and lifetime recall periods. The subsequent analyses were conducted in two stages. First, each form of IPV (physical, sexual, or emotional) and a composite indicator of any conventional IPV were considered as outcomes for both past 12-month and lifetime experiences. The two exposure variables, reproductive coercion pattern and contraceptive coercion typology, were entered separately into each model along with all covariates; the reference groups were women reporting no reproductive coercion and women reporting no contraceptive coercion, respectively. Survey-weighted Poisson regression models with robust standard errors were used because the outcomes were common, making prevalence ratios more appropriate and interpretable than odds ratios estimated from logistic regression models. Results are reported as adjusted prevalence ratios (aPRs) with corresponding 95% confidence intervals. At the second stage, I cross-classified conventional IPV and reproductive coercion, separately for past 12-month and lifetime experiences, to generate a four-category variable: neither, conventional IPV only, reproductive coercion only, and both. This variable was dichotomised to identify reproductive coercion without reported conventional IPV (women who reported reproductive coercion but did not report any of the physical, sexual, or emotional IPV items included in the survey) versus all other women (neither, conventional IPV only, or both). This category describes reproductive coercion not captured by the measured conventional IPV indicators; it does not indicate the absence of unmeasured or unreported violence. Survey-weighted multivariable logistic regression models, fitted separately for past 12-month and lifetime experiences, were used to examine associations between covariates and this outcome, with results reported as adjusted odds ratios (aORs) with 95% CIs. The unweighted numbers of events and non-events for each model were examined before interpretation, both overall and within each covariate category. Model estimates were checked against these counts. Multicollinearity was checked before running each model using the variance inflation factor (VIF). Finally, survey-weighted population extrapolations were calculated by multiplying survey-weighted prevalence by census population denominators from the 2022 Population and Housing Census: the past 12-month prevalence was applied to the currently married female population and the lifetime prevalence to the ever-married female population, nationally and by division. The 95% uncertainty intervals (UIs) were obtained by applying the lower and upper 95% confidence limits of the prevalence estimates to the same denominators and do not include uncertainty in the census counts. All analyses were conducted using Stata version 19 (StataCorp, College Station, TX, USA).

### Ethics statement

This study used deidentified secondary data from the Bangladesh Violence Against Women (VAW) Survey 2024, the third national survey of its kind, conducted by the Bangladesh Bureau of Statistics (BBS) in collaboration with the Ministry of Women and Children Affairs, with technical support from the United Nations Population Fund (UNFPA) Bangladesh, the UNFPA Asia and the Pacific Regional Office, and the University of Melbourne, Australia [4]. The survey was conducted by BBS, the national statistical organisation of Bangladesh, following international ethical and safety standards for research on violence against women [4]. The institutional review committee of the BBS review the survey protocol and approved the survey. Verbal informed consent was obtained from all respondents using a standardised script after they were informed about the purpose of the survey, confidentiality, and their right to decline, skip questions, or stop the interview at any time, and explicit permission was sought again before the violence modules [4]. This secondary analysis used a fully deidentified dataset obtained through BBS data access procedures, involved no contact with participants, and posed no additional risk to them.

## Results

### Background characteristics of the respondents

The background characteristics of the respondents are presented in Table 1. Among 24,951 ever-married women included in the analysis, 44.6% were aged ≥40 years and 73.6% had married before age 18 years. More than half (53.1%) had 1–2 living children, 50.5% had primary education, and only 4.1% reported earning money. Most respondents were Muslim (88.4%), while 40.9% reported at least some level of disability. Husbands commonly had primary education (51.6%), and 8.5% of women reported that their husband had an extramarital relationship. Most women resided in rural areas (70.4%) and non-slum households (97.7%). By wealth status, 49.0% belonged to the two poorest quintiles, and geographically the largest proportion lived in Dhaka division (28.6%), followed by Chattogram (18.4%).

**Table 1:** Background characteristics of respondents, Violence Against Women Survey 2024, Bangladesh (N = 24,951).

Characteristic	Weighted % (95% CI)
**Women’s age (years)**	
15–19	5.78 (5.24–6.37)
20–29	24.13 (22.90–25.40)
30–39	25.47 (24.62–26.34)
≥40	44.62 (43.39–45.85)
**Child marriage (marriage before 18 years)**	
No	26.43 (25.33–27.55)
Yes	73.57 (72.45–74.67)
**Number of living children**	
None	2.29 (1.98–2.65)
1–2	53.09 (51.81–54.37)
3–4	34.47 (33.35–35.61)
≥5	10.15 (9.49–10.84)
**Women’s education**	
Primary or less	50.45 (49.05–51.85)
Secondary	37.98 (36.78–39.19)
Higher secondary+	11.57 (10.76–12.44)
**Women’s employment status**	
No	95.86 (94.24–97.04)
Yes	4.14 (2.96–5.76)
**Religion**	
Muslim	88.39 (85.26–90.92)
Non-Muslim	11.61 (9.08–14.74)
**Disability status**	
None	59.11 (57.68–60.53)
Some difficulty	31.39 (30.22–32.57)
Moderate/severe difficulty	9.50 (8.85–10.19)
**Husband’s education**	
Primary or less	51.64 (50.26–53.01)
Secondary	31.43 (30.26–32.62)
Higher secondary+	16.93 (15.80–18.13)
**Husband extra-marital relationship**	
No	91.50 (90.74–92.20)
Yes	8.50 (7.80–9.26)
**Household type**	
Non-slum	97.72 (93.96–99.16)
Slum	2.28 (0.84–6.04)
**Wealth quintile**	
Poorest	23.47 (21.99–25.02)
Poorer	25.56 (24.23–26.93)
Middle	19.76 (18.52–21.05)
Richer	19.06 (17.34–20.90)
Richest	12.16 (11.03–13.39)
**Place of residence**	
Rural	70.39 (67.30–73.30)
Urban	29.61 (26.70–32.70)
**Administrative division**	
Barishal	5.01 (4.62–5.44)
Chattogram	18.40 (16.81–20.10)
Dhaka	28.61 (25.52–31.93)
Khulna	11.79 (10.24–13.54)
Mymensingh	7.54 (6.29–9.01)
Rajshahi	12.52 (11.14–14.04)
Rangpur	11.88 (10.63–13.27)
Sylhet	4.24 (3.69–4.87)

### Prevalence of intimate partner violence, reproductive coercion, reproductive coercion without reported conventional IPV, and number of violence domains

Among currently married women in Bangladesh, 27.3% (95% CI, 26.11–28.52) reported experiencing at least one form of conventional IPV in the past 12 months (Table 2). Emotional violence was the most common recent form of violence (19.6%), followed by physical violence (11.7%) and sexual violence (10.5%). Among ever-married women, 60.4% (95% CI, 59.13–61.66) reported experiencing at least one form of conventional IPV during their lifetime. Across the life course, physical violence was the most prevalent domain (47.3%), followed by emotional violence (37.8%) and sexual violence (32.6%).

**Table 2:** Prevalence of intimate partner violence, reproductive coercion, reproductive coercion without reported conventional IPV, and number of violence domains among currently married women (past 12 months) and ever-married women (lifetime) in Bangladesh, 2024.

Domain / Indicator	Past 12 months estimate among currently married women, % (95% CI)	Lifetime estimate among ever-married women, % (95% CI)
**Conventional IPV**		
Physical IPV	11.74 (11.06–12.45)	47.29 (46.06–48.53)
Emotional IPV	19.61 (18.52–20.75)	37.82 (36.59–39.08)
Sexual IPV	10.54 (9.82–11.31)	32.58 (31.33–33.87)
**Any conventional IPV**	**27.30 (26.11–28.52)**	**60.40 (59.13–61.66)**
**Reproductive coercion**		
Any contraceptive coercion	2.75 (2.42–3.13)	5.74 (5.23–6.31)
Forced contraceptive use	1.99 (1.71–2.32)	3.73 (3.32–4.19)
Prevented contraceptive use	1.05 (0.86–1.28)	2.57 (2.25–2.95)
Both forced and prevented use	0.29 (0.20–0.43)	0.56 (0.44–0.71)
Pregnancy-related coercion	1.16 (0.93–1.45)	4.73 (4.24–5.27)
**Any reproductive coercion**	**3.59 (3.19–4.05)**	**9.25 (8.50–10.06)**
**Combined prevalence**		
Any abuse (conventional IPV or reproductive coercion)	28.61 (27.39–29.87)	61.18 (59.89–62.45)
Both conventional IPV and reproductive coercion	2.28 (1.99–2.61)	8.48 (7.76–9.25)
Reproductive coercion without reported conventional IPV+	1.31 (1.08–1.59)	0.78 (0.60–1.01)
Number of violence domains experienced*		
None	71.39 (70.13–72.61)	38.82 (37.55–40.11)
One domain	16.50 (15.59–17.45)	21.21 (20.33–22.12)
Two domains	7.97 (7.36–8.63)	19.07 (18.22–19.95)
Three domains	3.53 (3.14–3.95)	15.98 (15.09–16.92)
Four domains	0.62 (0.48–0.80)	4.91 (4.44–5.43)
**Relative increase after adding reproductive coercion†**	**4.81%**	**1.29%**

Notes: Estimates are weighted and account for the complex survey design of the Bangladesh Violence Against Women Survey 2024. Conventional IPV includes physical, emotional, and sexual violence. Reproductive coercion includes contraceptive, pregnancy-related, or abortion-related coercion.

+ Women who reported reproductive coercion but did not report any of the physical, sexual, or emotional IPV items included in the survey. This does not indicate the absence of unmeasured or unreported violence.

* Violence domains include physical violence, emotional violence, sexual violence, and reproductive coercion.

† Relative increase in overall violence prevalence after adding reproductive coercion to conventional IPV. Relative increase calculated as: (Any abuse (violence or reproductive coercion)− any conventional IPV) ÷ any conventional IPV × 100.

Abbreviation: CI, confidence interval.

Among currently married women, 3.59% (95% CI, 3.19–4.05) reported experiencing reproductive coercion in the past 12 months. Overall, 2.28% (95% CI, 1.99–2.61) of currently married women reported both reproductive coercion and conventional IPV, while 1.31% (95% CI, 1.08–1.59) reported reproductive coercion without reporting any of the measured physical, sexual, or emotional IPV items. Contraceptive coercion was reported by 2.75% of currently married women, and pregnancy-related coercion by 1.16%. Overall, 28.61% (95% CI, 27.39–29.87) reported either conventional IPV or reproductive coercion in the past 12 months. Adding reproductive coercion to conventional IPV therefore increased the estimated past 12-month prevalence of any reported abuse by 1.31 percentage points, a relative increase of 4.81%.

Among ever-married women, 9.25% (95% CI, 8.50–10.06) reported experiencing reproductive coercion during their lifetime. Overall, 8.48% (95% CI, 7.76–9.25) of ever-married women reported both reproductive coercion and conventional IPV, while 0.78% (95% CI, 0.60–1.01) reported reproductive coercion without reporting any of the measured conventional IPV items. Lifetime prevalence was 5.74% for contraceptive coercion and 4.73% for pregnancy-related coercion. Overall, 61.18% (95% CI, 59.89–62.45) reported either conventional IPV or reproductive coercion during their lifetime. Adding reproductive coercion to conventional IPV increased the estimated lifetime prevalence of any reported abuse by 0.78 percentage points, a relative increase of 1.29%.

### Associations of reproductive coercion with conventional intimate partner violence indicators in Bangladesh

The associations of reproductive coercion pattern and contraceptive coercion typology with conventional IPV were examined using survey-weighted multivariable Poisson regression models. Full model results are presented in S1 Table–S16 Table, and summary estimates are shown in Fig 1 and 2. In the past 12 months, women reporting contraceptive coercion only had more than twice the prevalence of any conventional IPV (aPR 2.06, 95% CI 1.86–2.28), physical violence (aPR 2.22, 95% CI 1.85–2.66), and emotional violence (aPR 2.26, 95% CI 1.98–2.58), and more than three times the prevalence of sexual violence (aPR 3.16, 95% CI 2.62–3.82), compared with women reporting no reproductive coercion. Women reporting pregnancy-related coercion without contraceptive coercion likewise had a higher prevalence of any conventional IPV (aPR 1.77, 95% CI 1.42–2.20), physical violence (aPR 1.56, 95% CI 1.06–2.29), emotional violence (aPR 1.76, 95% CI 1.33–2.33), and sexual violence (aPR 2.51, 95% CI 1.79–3.52). The strongest associations were observed among women exposed to both contraceptive and pregnancy-related coercion, with higher prevalence of any conventional IPV (aPR 2.74, 95% CI 2.26–3.33), physical violence (aPR 3.42, 95% CI 2.25–5.19), emotional violence (aPR 3.45, 95% CI 2.66–4.47), and sexual violence (aPR 4.85, 95% CI 3.45–6.83). Similar patterns were observed for lifetime exposure. Women reporting contraceptive coercion only had a higher lifetime prevalence of any conventional IPV (aPR 1.36, 95% CI 1.29–1.43), physical violence (aPR 1.37, 95% CI 1.28–1.46), emotional violence (aPR 1.64, 95% CI 1.53–1.76), and sexual violence (aPR 1.96, 95% CI 1.79–2.15). Women reporting pregnancy-related coercion without contraceptive coercion also had a higher lifetime prevalence of any conventional IPV (aPR 1.58, 95% CI 1.53–1.64), physical violence (aPR 1.43, 95% CI 1.33–1.54), emotional violence (aPR 1.70, 95% CI 1.57–1.83), and sexual violence (aPR 3.12, 95% CI 2.94–3.31). Women experiencing both contraceptive and pregnancy-related coercion had a higher lifetime prevalence of any conventional IPV (aPR 1.53, 95% CI 1.47–1.60), physical violence (aPR 1.52, 95% CI 1.38–1.68), emotional violence (aPR 1.84, 95% CI 1.68–2.03), and sexual violence (aPR 2.95, 95% CI 2.74–3.17).

**Fig 1:**
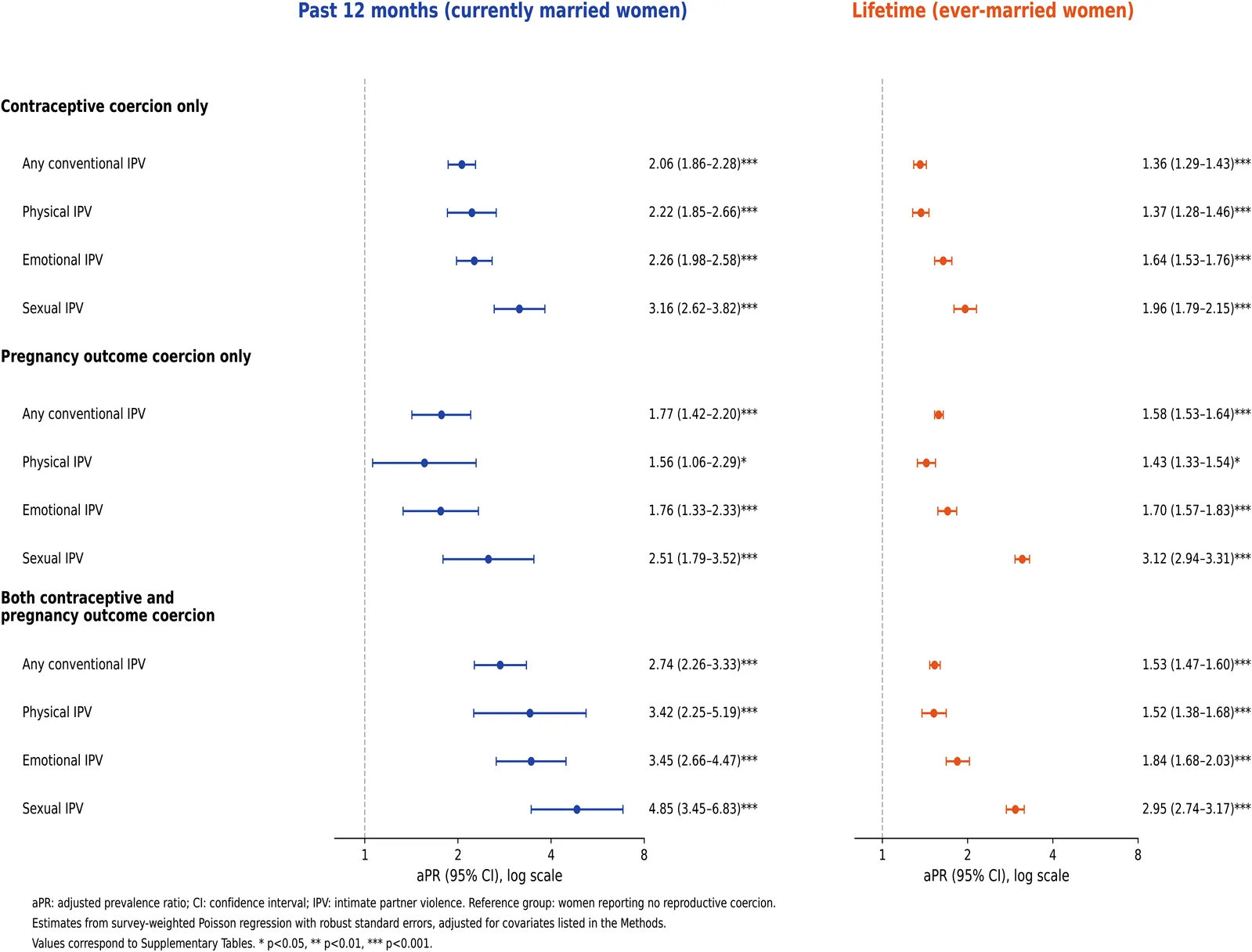
Adjusted prevalence ratios (aPRs) for conventional IPV outcomes (past 12 months among currently married women and lifetime among ever-married women) by pattern of reproductive coercion in Bangladesh, 2024.

**Fig 2:**
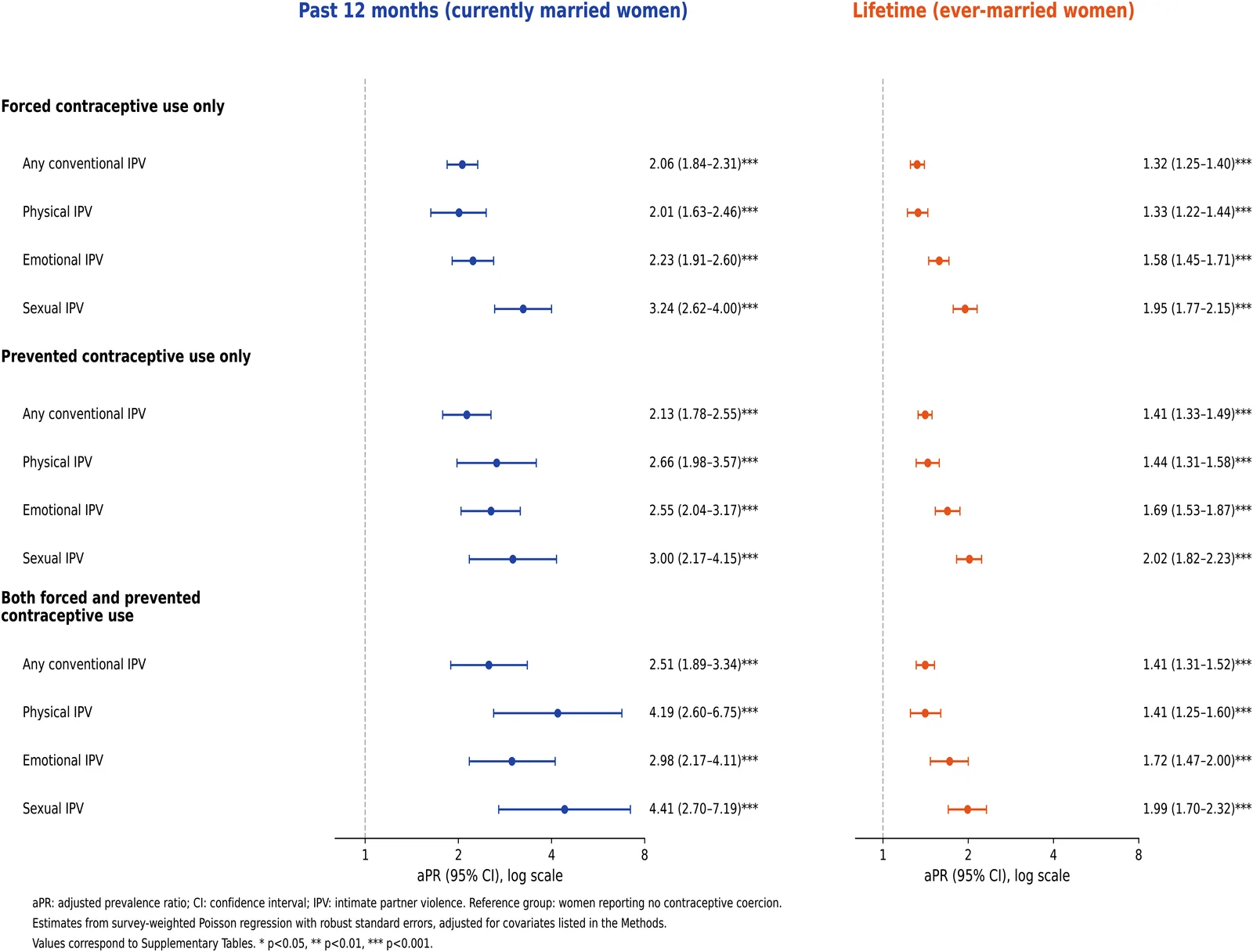
Adjusted prevalence ratios (aPRs) for conventional IPV outcomes (past 12 months among currently married women and lifetime among ever-married women) by pattern of contraceptive coercion in Bangladesh, 2024.

Note: Full model results are presented in S1 Table–S4 Tables (past 12 months) and 9–12 (lifetime).

When contraceptive coercion was disaggregated, all forms were associated with a higher prevalence of conventional IPV. In the past 12 months, women reporting forced contraceptive use only had a higher prevalence of any conventional IPV (aPR 2.06, 95% CI 1.84–2.31), physical violence (aPR 2.01, 95% CI 1.63–2.46), emotional violence (aPR 2.23, 95% CI 1.91–2.60), and sexual violence (aPR 3.24, 95% CI 2.62–4.00). Women reporting prevented contraceptive use only had similarly higher prevalence of any conventional IPV (aPR 2.13, 95% CI 1.78–2.55), physical violence (aPR 2.66, 95% CI 1.98–3.57), emotional violence (aPR 2.55, 95% CI 2.04–3.17), and sexual violence (aPR 3.00, 95% CI 2.17–4.15). The strongest associations were observed among women reporting both forced and prevented contraceptive use, with higher prevalence of any conventional IPV (aPR 2.51, 95% CI 1.89–3.34), physical violence (aPR 4.19, 95% CI 2.60–6.75), emotional violence (aPR 2.98, 95% CI 2.17–4.11), and sexual violence (aPR 4.41, 95% CI 2.70–7.19).

Similar patterns were observed for lifetime exposure. Women reporting forced contraceptive use only had a higher lifetime prevalence of any conventional IPV (aPR 1.32, 95% CI 1.25–1.40), physical violence (aPR 1.33, 95% CI 1.22–1.44), emotional violence (aPR 1.58, 95% CI 1.45–1.71), and sexual violence (aPR 1.95, 95% CI 1.77–2.15). Women reporting prevented contraceptive use only also had a higher prevalence of any conventional IPV (aPR 1.41, 95% CI 1.33–1.49), physical violence (aPR 1.44, 95% CI 1.31–1.58), emotional violence (aPR 1.69, 95% CI 1.53–1.87), and sexual violence (aPR 2.02, 95% CI 1.82–2.23). Among women reporting both forced and prevented contraceptive use, the corresponding aPRs were 1.41 (95% CI 1.31–1.52) for any conventional IPV, 1.41 (95% CI 1.25–1.60) for physical violence, 1.72 (95% CI 1.47–2.00) for emotional violence, and 1.99 (95% CI 1.70–2.32) for sexual violence.

Note: Full model results are presented in S5 Table–S8 Tables (past 12 months) and 13–16 (lifetime).

### Distribution of joint exposure to conventional intimate partner violence and reproductive coercion

The distribution of joint exposure to conventional IPV and reproductive coercion is presented in S17 Table. Across all subgroups, the dominant pattern in the past 12 months was absence of both conventional IPV and reproductive coercion (>70%), whereas lifetime estimates showed a clear shift towards higher exposure, with conventional IPV alone exceeding 50% in many groups. Joint exposure remained relatively uncommon in the past 12 months (generally ≤6%) but increased notably over the lifetime, particularly among younger women and those with no children. Reproductive coercion without reported conventional IPV was uncommon in both periods (<1%). The most pronounced disparities were observed among women whose husbands had extra-marital relationships, where lifetime exposure to conventional IPV (74.53%) and joint exposure (19.85%) were substantially higher than recent estimates. Higher lifetime burden was also evident among women with child marriage, lower education, disability, and lower wealth. Differences by residence were minimal, with modest regional variation.

### Distribution of reproductive coercion without reported conventional intimate partner violence across respondent characteristics

The distribution of reproductive coercion without reported conventional IPV across the characteristics examined is presented in S18 Table. Prevalence was low across all subgroups in both recall periods: 1.31% in the past 12 months and 0.78% over the lifetime. In the past 12 months, prevalence was highest among adolescents aged 15–19 years (3.02%) and lowest among women aged 40 years or older (0.44%). Estimates by age group for both recall periods are shown in Fig 3. Point estimates in the past 12 months were somewhat higher among women with secondary education (1.67%), Muslim women (1.23%), and women whose husbands had secondary education (1.49%), and lower among women with moderate or severe functional difficulty (0.54%) and non-Muslim women (0.53%), although many confidence intervals overlapped. Lifetime estimates were low across all groups, with no clear socioeconomic gradient.

**Fig 3:**
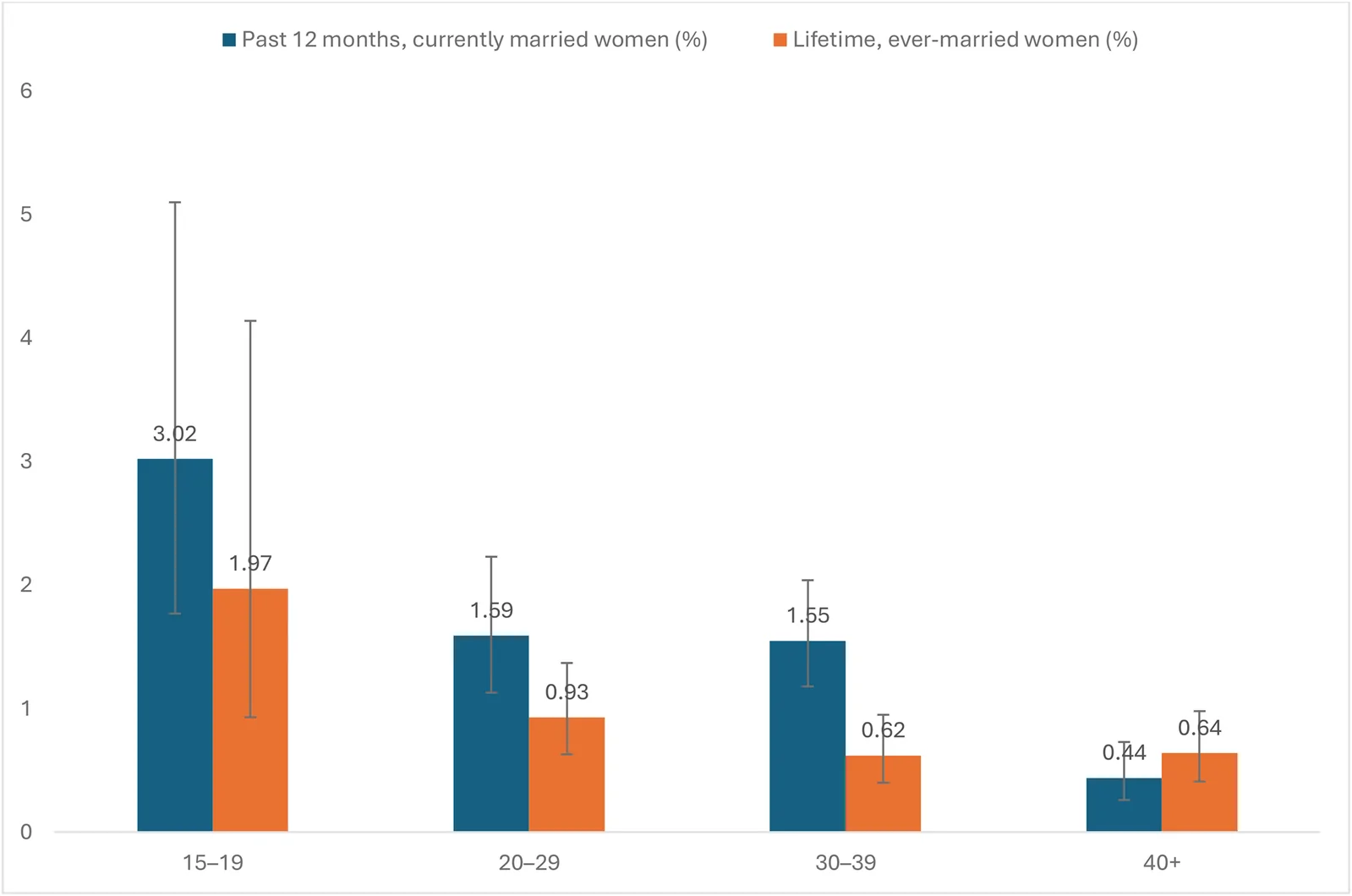
Prevalence of reproductive coercion without reported conventional intimate partner violence by age group among currently married women (past 12 months) and ever-married women (lifetime) in Bangladesh, 2024.

### Factors associated with reproductive coercion without reported conventional intimate partner violence in Bangladesh

Factors associated with reproductive coercion without reported conventional intimate partner violence were explored through multivariate logistic regression models and the results are presented in S19 Table. In the past 12 months, women with some functional difficulty had higher odds of reporting reproductive coercion without conventional IPV (aOR 1.84, 95% CI 1.26–2.70), whereas women with five or more living children had lower odds (aOR 0.08, 95% CI 0.02–0.40). Respondents in Chattogram division had higher odds than those in Barishal division (aOR 2.09, 95% CI 1.07–4.07). Over the lifetime, women whose husbands had an extra-marital relationship had markedly lower odds (aOR 0.01, 95% CI 0.001–0.04).

### Survey-weighted population extrapolations of reproductive coercion without reported conventional IPV in Bangladesh

Survey-weighted population extrapolations were calculated by multiplying survey-weighted prevalence by census population denominators from the 2022 Population and Housing Census. Because past 12-month experiences were analysed only among currently married women, the past 12-month prevalence was applied to the currently married female population, whereas the lifetime prevalence was applied to the ever-married female population. Using this approach, an estimated 645,483 (95% UI, 532,154–783,449) currently married women reported reproductive coercion without reported conventional IPV in the past 12 months, and 437,414 (95% UI 337,106–567,462) ever-married women did so over the lifetime (Table 3). In the past 12 months, an estimated 508,876 (385,865–632,824) women reported contraceptive coercion without reported conventional IPV. Overall, 35.76% (95% CI 30.74–40.77) of women reporting reproductive coercion in the past 12 months and 8.41% (95% CI 6.36–10.47) over the lifetime did not report any of the measured physical, sexual, or emotional IPV items. These figures estimate how many women reported reproductive coercion that would not be reflected in prevalence estimates based only on these IPV items; they do not indicate whether these women were identified by, or had contact with, services or other systems.

**Table 3:** Survey-weighted population extrapolations of reproductive coercion without reported conventional IPV among currently married women (past 12 months) and ever-married women (lifetime) in Bangladesh, 2024.

Indicator	Past 12 months (currently married women)	Lifetime (ever-married women)
Population denominator, 2022 Population and Housing Census	Currently married women: 49,273,511	Ever-married women: 56,184,352
Survey-weighted prevalence of reproductive coercion without reported conventional IPV, % (95% CI)	1.31 (1.08–1.59)	0.78 (0.60–1.01)
Estimated number of women with reproductive coercion without reported conventional IPV (95% UI)	645,483 (532,154–783,449)	437,414 (337,106–567,462)
Estimated number of women with contraceptive coercion without reported conventional IPV (95% UI)	508,876 (385,865 −632,824)	437,414 (308,232-565,629)
Share of women reporting reproductive coercion who did not report conventional IPV, % (95% CI)	35.76 (30.74–40.77)	8.41 (6.36–10.47)

**Notes:** Estimated numbers are survey-weighted population extrapolations, calculated as survey-weighted prevalence multiplied by the census population denominator. Past 12 months: 1.31% × [[49,273,511]] currently married women ([[2022 Population and Housing Census, table reference; age range]]) = 645,483. Lifetime: 0.78% × 56,184,352 ever-married women (2022 Population and Housing Census) = 437,414; unrounded prevalence estimates were used in the calculations. The 95% uncertainty intervals (UIs) apply the lower and upper 95% confidence limits of each prevalence estimate to the same denominator and do not include uncertainty in census counts. The share not captured refers to the proportion of women reporting reproductive coercion who did not report any of the measured physical, sexual, or emotional IPV items; it does not indicate the absence of unmeasured or unreported violence. Conventional IPV includes physical, emotional, or sexual violence by a husband.

The estimated number of women reporting reproductive coercion without reported conventional IPV varied across divisions (Fig 4). These absolute numbers reflect both divisional prevalence (S18 Table) and the size of each division's population, so a division with a large population can have a high number despite a relatively low prevalence. In the past 12 months, the highest estimated numbers were observed in Chattogram (172,148), followed by Khulna (101,644), Dhaka (97,977), and Rajshahi (96,314). For lifetime experience, Dhaka had the largest number (89,137), followed by Mymensingh (76,237), Chattogram (75,854), and Rajshahi (68,848), although Dhaka had the lowest lifetime prevalence of all divisions (0.55%) and Mymensingh the highest (1.80%). Barishal and Sylhet, the two divisions with the smallest populations, had the lowest numbers in both periods. Divisional prevalence estimates were imprecise, with overlapping confidence intervals.

**Fig 4:**
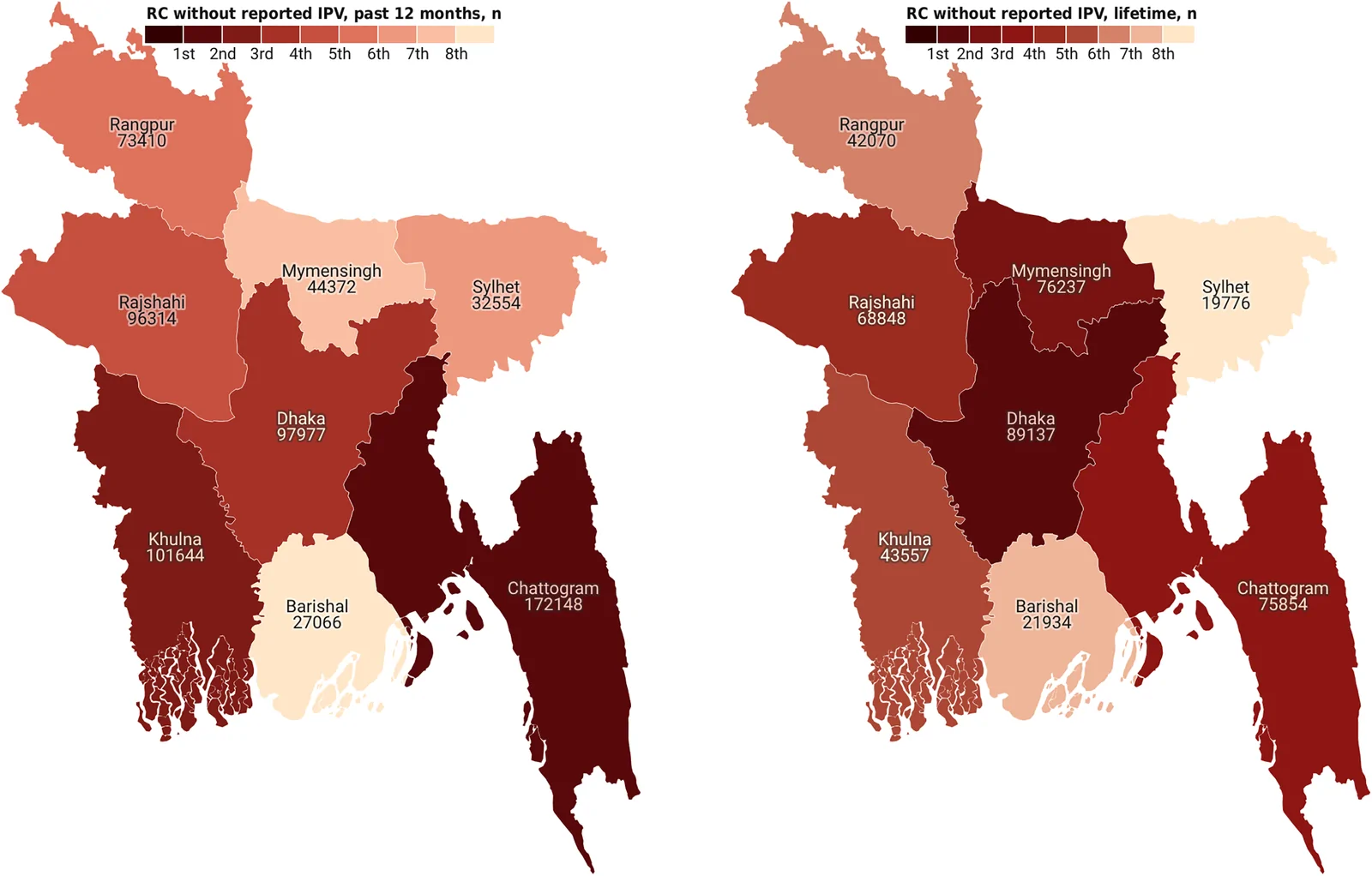
Estimated number of women reporting reproductive coercion without reported conventional IPV, by administrative division, Bangladesh, 2024 (past 12 months among currently married women and lifetime among ever-married women). (The map was generated by the author using ArcGIS version 10. The base-map shapefile was sourced from The Humanitarian Data Exchange (https://data.humdata.org/dataset/cod-ab-bgd) and is licensed under the Creative Commons Attribution for Intergovernmental Organisations (CC BY-IGO) license. The data have been adapted by the author, and the licensor does not endorse this work).

## Discussion

This study examined the associations between reproductive coercion and the physical, sexual, and emotional IPV measured in a national survey, and estimated how many women reported reproductive coercion without reporting any of these measured forms of IPV. Although reproductive coercion was less prevalent than conventional IPV (3.59% vs 27.30% in the past 12 months; 9.25% vs 60.40% over the lifetime), its inclusion increased the prevalence of any reported abuse from 27.30% to 28.61% in the past 12 months and from 60.40% to 61.18% over the lifetime. Overall, 1.31% of currently married women in the past 12 months and 0.78% of ever-married women over the lifetime reported reproductive coercion but none of the physical, sexual, or emotional IPV items included in the survey. Applied to census population counts, these proportions correspond to approximately 645,483 and 437,414 women, respectively, whose reported reproductive coercion would not be reflected in prevalence estimates based only on these IPV items. Reproductive coercion was also consistently associated with a higher prevalence of each measured form of conventional IPV, with the largest aPRs among women reporting more than one form of coercion. Together, these findings indicate a measurement gap: survey instruments that omit reproductive coercion items do not capture this dimension of partner abuse. In Bangladesh, where national prevalence estimates inform policy priorities, such an omission may lead to reproductive coercion being under-represented in policy and programme planning. However, the data do not show whether these women were identified by health, legal, or social services, and the cross-sectional design cannot establish the relative importance of reproductive coercion compared with other forms of violence.

These findings should be situated within the growing international literature on reproductive coercion, which was initially developed largely through clinical and population-based research in the United States before being extended to diverse international settings [14,22,23]. Using data from the nationally representative US National Intimate Partner and Sexual Violence Survey, Basile and colleagues estimated that 8.4% of women had experienced reproductive coercion by an intimate partner during their lifetime [22]. This estimate is of a similar magnitude to the 9.25% lifetime prevalence observed in our study, despite substantial differences in context, survey instruments, and item wording, suggesting that reproductive coercion is not unique to Bangladesh or South Asia. Evidence from sub-Saharan Africa indicates considerable variation across settings. In longitudinal population-based cohorts, for example, past-year reproductive coercion prevalence in Uganda was 15.8% at baseline and 17.8% at follow-up [24]. Such variation is consistent with international evidence showing that estimates of reproductive coercion differ across settings and study populations, potentially reflecting differences in social and gender relations, contraceptive contexts, populations studied, and measurement approaches [14,23,24]. Consistent with our finding that reproductive coercion frequently co-occurs with, rather than substitutes for, other forms of partner violence, previous international research has documented substantial intersections between reproductive coercion and intimate partner violence [14,23]. Evidence also suggests that its consequences may extend beyond the immediate coercive experience to subsequent reproductive behaviours. In Burkina Faso, for example, women reporting past-year reproductive coercion had a substantially higher risk of subsequent contraceptive discontinuation than continuation [24]. Taken together, these comparisons suggest that the patterns observed in Bangladesh, particularly the co-occurrence of reproductive coercion with conventional forms of violence and the presence of a smaller subgroup reporting reproductive coercion without other measured violence, are broadly consistent with international evidence. However, the proportion of reproductive coercion that is not captured by standard physical, sexual, and emotional IPV items is likely to be context-dependent and should not be extrapolated beyond Bangladesh without further comparative research.

The co-occurrence of reproductive coercion and conventional IPV indicates that these forms of abuse frequently occur together in this population. Women reporting reproductive coercion had a higher prevalence of each measured form of IPV, with the largest aPRs among those reporting more than one form of coercion, consistent with a graded association. This clustering is consistent with evidence, largely from high-income settings, showing that reproductive coercion is embedded within broader dynamics of intimate partner violence and reflects gendered power imbalances and control over women’s bodies and reproductive decision-making [25,26]. In LMIC contexts, including Bangladesh, this overlap may be shaped by structural and normative factors such as patriarchal gender norms, son preference, and strong expectations around fertility and family formation, which may legitimise control over women’s reproductive choices [27]. Within the coercive control framework, the observed pattern is consistent with reproductive coercion operating as one of several tactics through which husbands may exert control, with control over contraception and pregnancy occurring alongside, or potentially being enforced through, physical, emotional, or sexual violence. In Bangladesh, where marriage often occurs early, fertility expectations are strong, and decisions about contraception and childbearing may involve husbands and extended family members, women's reproductive autonomy may be constrained both within the couple and by wider household norms. The finding that some women reported reproductive coercion without the other measured forms of IPV partly complicates this framework, which would predict clustering of coercive tactics; this pattern may reflect under-reporting, controlling behaviours or economic violence that were not included in the conventional IPV definition, or reproductive coercion occurring with fewer co-occurring tactics. The survey did not measure the intent behind these behaviours, women's fear, or the underlying dynamics of power and control, so these data are consistent with, but do not test, the coercive control framework, and the mechanisms linking reproductive coercion to other forms of IPV cannot be determined from this analysis. Previous studies suggest that failure to recognise the co-occurrence of reproductive coercion and other forms of violence may lead to incomplete identification and management of abuse, undermine family planning services, and reduce the effectiveness of violence prevention and response strategies [23]. This study did not measure unintended pregnancy, forced continuation or termination of pregnancy, unsafe abortion, mental health, access to care, or women's own experiences of reproductive autonomy, so these consequences reflect previous literature rather than outcomes demonstrated here. In addition, improving identification alone is unlikely to reduce reproductive coercion because the behaviour is embedded in unequal gender relations and socio-cultural norms surrounding marriage, fertility, and women's reproductive decision-making. Previous literature suggests that an effective response requires coordinated multisectoral action that extends beyond healthcare to include community programmes that challenge inequitable gender norms, engagement of men and families, legal protection against coercive behaviours, women's empowerment initiatives, accessible social support and referral services, and collaboration with legal aid services, social protection programmes, community and religious leaders, and women's rights organisations [10,11,14]. Given documented inequalities in healthcare access across Bangladesh's rural, urban, and informal settlement populations, a response centred solely on clinical settings may not reach the women who are most structurally disadvantaged. Inclusion of reproductive coercion in national surveys and routine identification in services should therefore be considered components of a broader multisectoral strategy rather than ends in themselves.

Alongside this overlap, a subgroup of women reported reproductive coercion without reporting any of the physical, sexual, or emotional IPV items included in the survey. This classification reflects what was measured and reported in a single interview. It does not indicate that these women were free from other forms of partner violence, because unmeasured forms of abuse, including controlling behaviours and economic violence that were not part of the conventional IPV definition used in the survey, and under-reporting of physical, sexual, or emotional IPV could also place women in this category. I therefore interpret this group as reproductive coercion not captured by the measured conventional IPV indicators, rather than as a distinct population experiencing reproductive coercion in isolation from other abuse. Although this group represents a small proportion of women, survey-weighted population extrapolations suggest that it corresponds to several hundred thousand women in Bangladesh, consistent with the public health principle that low-prevalence exposures can involve large absolute numbers in populous settings [28]. In addition, the survey primarily focused on conventional IPV measures and did not include comprehensive measures of reproductive coercion and coercive control, such as the broader set of items recently recommended for global monitoring, which may lead to underestimation of reproductive coercion overall and of reproductive coercion not captured by the measured IPV indicators [29]. Whether women in this group are identified or supported by violence response or reproductive health services could not be assessed, because the survey data used here did not link reproductive coercion to disclosure, screening, referral, or service contact; this remains a hypothesis requiring further research. Because policy priorities in many LMICs are informed by survey-based prevalence estimates, omitting reproductive coercion items may lead to its under-representation in national strategies, underscoring the value of including validated items in future surveys.

The administrative divisions of Bangladesh differ substantially in population size, urbanisation, socioeconomic development, and access to health and social services. The estimated numbers of women reporting reproductive coercion without reported conventional IPV varied across divisions, but this variation reflects both differences in prevalence and differences in population size. For example, Dhaka had the largest lifetime number despite the lowest lifetime prevalence, whereas Mymensingh had the highest lifetime prevalence, and divisional prevalence estimates had overlapping confidence intervals. Differences between divisions in the prevalence of this outcome and in the prevalence of conventional IPV may reflect differences in reporting, measurement, or social context, but the reasons could not be examined in this analysis [4]. Previous evidence from Bangladesh and other LMICs suggests that regional differences in gender norms, service access, and economic opportunities may shape both the occurrence and reporting of violence [3,4,30]. Processes such as urbanisation, migration, and changing labour dynamics may also influence household power relations and reproductive expectations; however, none of these contextual factors was measured in this study, and these explanations should be regarded as hypotheses. Subnational estimates may nevertheless help identify where further research and service planning are needed, because national averages can mask variation between divisions.

The proportion of women reporting reproductive coercion who did not report any measured conventional IPV was higher for the past 12 months than for the lifetime, whereas lifetime reproductive coercion more often co-occurred with conventional IPV. Because these data are cross-sectional and the two recall periods refer to different populations (currently married and ever-married women), this difference cannot be interpreted as evidence that reproductive coercion precedes, or progresses into, other forms of IPV within relationships. It may instead reflect age or cohort differences, recall of past events, differences between the reference populations, measurement, or the longer period over which lifetime IPV can accumulate. Whether reproductive coercion precedes other forms of abuse, including in the context of early marriage and fertility expectations in Bangladesh, remains a theoretical possibility that was not tested in this study and requires longitudinal research [31,32].

Few socio-demographic characteristics were associated with reproductive coercion without reported conventional IPV in the exploratory models, suggesting that this pattern is not confined to groups commonly identified as being at higher risk of conventional IPV. This interpretation should be considered alongside the small number of events, which limited statistical power, and the widespread distribution of characteristics such as rural residence, child marriage, and lower educational attainment in Bangladesh. In contrast to conventional IPV, which is often more common among women experiencing poverty, lower education, or early marriage, this pattern appeared across diverse population groups with limited clustering [3,4]. Similarly, the absence of a clear difference between slum and non-slum households suggests that this pattern is not explained solely by material deprivation, although the number of women living in slum households was small. The characteristics examined are also relevant to women's circumstances beyond their role as covariates. Child marriage and a larger number of children may reflect longer exposure to marital and fertility expectations; education and employment may be associated with access to information, resources, and decision-making; and wealth and place of residence may shape access to contraception and services. Educational attainment was measured as the highest level of schooling and should not be interpreted as a measure of literacy. The higher odds among women with some functional difficulty may reflect several possibilities that were not examined in this study, including dependency on partners or family members, communication barriers, reduced access to contraception and health services, and constraints on disclosure and help-seeking, each of which may affect reproductive autonomy. The lower odds among women with five or more children and among women whose husbands had an extra-marital relationship may indicate that reproductive coercion in these circumstances more often co-occurs with reported conventional IPV, but both estimates were based on very few events and may be affected by sparse-data bias. These are possible interpretations rather than mechanisms established by this study.

Taken together, these findings support including validated measures of reproductive coercion alongside physical, sexual, and emotional IPV items in national violence surveys, so that prevalence estimates reflect this dimension of partner abuse. They also suggest a need to evaluate whether screening, counselling, and referral for reproductive coercion within family planning and maternal health services, together with broader multisectoral action involving legal and social protection, community initiatives that address inequitable gender norms, and interventions that strengthen women's reproductive decision-making, reach women who report reproductive coercion. Research on disclosure, service contact, and women's own experiences of reproductive autonomy is needed to determine whether women who report reproductive coercion without other measured IPV are reached by existing services.

### Strengths and limitations

This study has several strengths alongside some limitations. First, it uses nationally representative data from the Bangladesh Violence Against Women Survey 2024, enabling population-level estimates and subnational comparisons. The predominance of respondents residing in rural and non-slum households reflects the underlying population distribution of Bangladesh rather than disproportionate sampling, as all estimates accounted for the survey's complex sampling design and weighting procedures. Second, the analysis examines reproductive coercion alongside conventional IPV and estimates the proportion of women who reported reproductive coercion without reporting the physical, sexual, or emotional IPV items included in the survey. Third, survey-weighted population extrapolations with uncertainty intervals translate prevalence into approximate absolute numbers, which may be useful for planning in a highly populous setting. Fourth, the study captures contraceptive and pregnancy-related forms of reproductive coercion and examines their co-occurrence with different types of IPV. Finally, all models accounted for the complex survey design and a consistent, conceptually specified set of individual, partner, household, and geographic covariates. Several limitations should be considered when interpreting these findings. First, the cross-sectional design precludes causal inference, and neither the temporal ordering of reproductive coercion and conventional IPV nor progression between them can be established. Second, all measures are self-reported. Despite the survey's privacy procedures, including interviews by trained female enumerators, rescheduling when privacy could not be ensured, and a decoy questionnaire, IPV and reproductive coercion may have been under-reported because of stigma, fear, social desirability, recall, or interviewer effects. This limitation is directly relevant to the main classification: women who under-reported physical, sexual, or emotional IPV would be classified as reporting reproductive coercion without conventional IPV. Third, the survey was primarily designed to measure conventional IPV and did not include a comprehensive, validated set of reproductive coercion or coercive control items, and the conventional IPV definition used here did not include controlling behaviours or economic violence. As a result, reproductive coercion may be underestimated, and women classified as reporting reproductive coercion without conventional IPV may have experienced other unmeasured or unreported abuse; this group should therefore be interpreted as reproductive coercion not captured by the measured conventional IPV indicators. Fourth, the analysis was restricted to ever-married women because the relevant reproductive and sexual health information was collected only from this population, and past 12-month analyses were restricted to currently married women. The findings therefore cannot be generalised to never-married women. Fifth, the logistic regression models were based on a small number of outcome events, and some estimates were extreme or imprecise; these results are exploratory and may be affected by sparse-data bias. Sixth, the representativeness of the sample composition against external census benchmarks was not formally assessed beyond the survey's design-based weighting. The population extrapolations also assume that survey-weighted prevalence applies to census population counts, rely on census denominators whose uncertainty is not reflected in the uncertainty intervals, and do not indicate whether women were identified by services. Seventh, the survey did not separately identify migrant, internally displaced, or ethnic minority women, and the extent to which the findings apply to these populations could not be assessed. Eighth, the survey did not collect information on disclosure, service contact, fear, the intent behind coercive behaviours, or reproductive health outcomes in relation to reproductive coercion, so these could not be examined. Finally, reproductive coercion was categorised by domain (contraceptive versus pregnancy-related) rather than according to pregnancy-preventing versus pregnancy-promoting intent, a distinction emphasised in some qualitative literature as more closely aligned with coercive control dynamics. Despite these limitations, the use of nationally representative, survey-weighted data provides evidence on the prevalence and distribution of reproductive coercion among ever-married women in Bangladesh and on the extent to which it is reported alongside, or without, the IPV forms measured in standard instruments.

## Conclusion

Reproductive coercion is an under-recognised dimension of VAW in Bangladesh. It was strongly associated with reported physical, sexual, and emotional IPV, and a small proportion of women, corresponding to several hundred thousand women nationally, reported reproductive coercion without reporting any of the conventional IPV items included in the survey. This group reflects reproductive coercion not captured by the measured conventional IPV indicators and should not be interpreted as women free from other forms of violence. Few socio-demographic characteristics were associated with this pattern in exploratory analyses. These findings support integrating validated measures of reproductive coercion into national violence surveys. Further research is needed on disclosure, service contact, and women's experiences of reproductive autonomy to inform screening, counselling, and referral within family planning and maternal health services and broader gender-based violence programmes.

## Supporting information

S1 Table Association between experience of any conventional IPV within the past 12 months and patterns of reproductive coercion among currently married women in Bangladesh, 2024. (DOCX)

S2 Table Association between experience of any physical violence within the past 12 months and patterns of reproductive coercion among currently married women in Bangladesh, 2024. (DOCX)

S3 Table Association between experience of any emotional violence within the past 12 months and patterns of reproductive coercion among currently married women in Bangladesh, 2024. (DOCX)

S4 Table Association between experience of any sexual violence within the past 12 months and patterns of reproductive coercion among currently married women in Bangladesh, 2024. (DOCX)

S5 Table Association between experience of any conventional IPV within the past 12 months and pattern of contraceptive coercion among currently married women in Bangladesh, 2024. (DOCX)

S6 Table Association between experience of any physical violence within the past 12 months and pattern of contraceptive coercion among currently married women in Bangladesh, 2024. (DOCX)

S7 Table Association between experience of any emotional violence within the past 12 months and pattern of contraceptive coercion among currently married women in Bangladesh, 2024. (DOCX)

S8 Table Association between experience of any sexual violence within the past 12 months and pattern of contraceptive coercion among currently married women in Bangladesh, 2024. (DOCX)

S9 Table Association between experience of any conventional IPV over the lifetime and patterns of reproductive coercion among ever-married women in Bangladesh, 2024. (DOCX)

S10 Table Association between experience of any physical violence over the lifetime and patterns of reproductive coercion among ever-married women in Bangladesh, 2024. (DOCX)

S11 Table Association between experience of any emotional violence over the lifetime and patterns of reproductive coercion among ever-married women in Bangladesh, 2024. (DOCX)

S12 Table Association between experience of any sexual violence over the lifetime and patterns of reproductive coercion among ever-married women in Bangladesh, 2024. (DOCX)

S13 Table Association between experience of any conventional IPV over the lifetime and patterns of contraception coercion among ever-married women in Bangladesh, 2024. (DOCX)

S14 Table Association between experience of any physical violence over the lifetime and patterns of contraception coercion among ever-married women in Bangladesh, 2024. (DOCX)

S15 Table Association between experience of any emotional violence over the lifetime and patterns of contraception coercion among ever-married women in Bangladesh, 2024. (DOCX)

S16 Table Association between experience of any sexual violence over the lifetime and patterns of contraception coercion among ever-married women in Bangladesh, 2024. (DOCX)

S17 Table Distribution of joint exposure to conventional IPV and reproductive coercion by respondent characteristics among ever-married women in Bangladesh, 2024. (DOCX)

S18 Table Prevalence of reproductive coercion without reported conventional IPV by respondent characteristics among currently married women (past 12 months) and ever-married women (lifetime) in Bangladesh, 2024. (DOCX)

S19 Table Factors associated with reproductive coercion without reported conventional intimate partner violence among currently married women (past 12 months) and ever-married women (lifetime) in Bangladesh, 2024: exploratory survey-weighted logistic regression models. (DOCX)
