# Supplementary material for: Beyond conventional indicators: Reproductive coercion and its overlap with measured intimate partner violence in Bangladesh
Source: PLoS One. 2026 Oct 21;21(10):e0360372. doi: 10.1371/journal.pone.0360372 (PMC13686496; doi:10.1371/journal.pone.0360372)
Supplement: Supplementary file 2 [file pone.0360372.s002.docx]

Supplementary Table 2. Association between experience of any physical violence within the past 12 months and patterns of reproductive coercion among currently married women in Bangladesh, 2024

| **Characteristic** | **aPR** | **95% CI** | **p-value** |
| --- | --- | --- | --- |
| **Reproductive coercion pattern** |  |  |  |
| No reproductive coercion | 1.00 | Reference | — |
| Contraceptive coercion only | 2.22 | 1.85–2.66 | <0.001 |
| Pregnancy outcome coercion only | 1.56 | 1.06–2.29 | 0.024 |
| Both contraceptive and pregnancy outcome coercion | 3.42 | 2.25–5.19 | <0.001 |
| **Women’s age (years)** |  |  |  |
| 15–19 | 1.00 | Reference | — |
| 20–29 | 0.95 | 0.72–1.25 | 0.706 |
| 30–39 | 0.79 | 0.60–1.05 | 0.101 |
| ≥40 | 0.54 | 0.40–0.73 | <0.001 |
| **Child marriage (marriage before 18 years)** |  |  |  |
| No | 1.00 | Reference | — |
| Yes | 0.94 | 0.82–1.08 | 0.387 |
| **Number of living children** |  |  |  |
| None | 1.00 | Reference | — |
| 1–2 | 1.18 | 0.79–1.76 | 0.415 |
| 3–4 | 1.26 | 0.83–1.91 | 0.276 |
| ≥5 | 1.19 | 0.72–1.97 | 0.492 |
| **Women’s education** |  |  |  |
| Primary or less | 1.00 | Reference | — |
| Secondary | 0.89 | 0.77–1.03 | 0.120 |
| Higher secondary+ | 0.80 | 0.63–1.01 | 0.061 |
| **Women’s employment status** |  |  |  |
| No | 1.00 | Reference | — |
| Yes | 0.95 | 0.64–1.40 | 0.791 |
| **Religion** |  |  |  |
| Muslim | 1.00 | Reference | — |
| Non-Muslim | 1.11 | 0.90–1.37 | 0.323 |
| **Disability status** |  |  |  |
| None | 1.00 | Reference | — |
| Some difficulty | 1.19 | 1.05–1.34 | 0.005 |
| Moderate/severe difficulty | 1.31 | 1.07–1.61 | 0.009 |
| **Husband’s education** |  |  |  |
| Primary or less | 1.00 | Reference | — |
| Secondary | 0.96 | 0.86–1.08 | 0.499 |
| Higher secondary+ | 0.76 | 0.63–0.92 | 0.005 |
| **Husband extra-marital relationship** |  |  |  |
| No | 1.00 | Reference | — |
| Yes | 2.15 | 1.84–2.50 | <0.001 |
| **Household type** |  |  |  |
| Non-slum | 1.00 | Reference | — |
| Slum | 1.09 | 0.76–1.57 | 0.643 |
| **Wealth quintile** |  |  |  |
| Poorest | 1.00 | Reference | — |
| Poorer | 0.91 | 0.79–1.05 | 0.191 |
| Middle | 0.92 | 0.77–1.10 | 0.366 |
| Richer | 0.86 | 0.71–1.03 | 0.100 |
| Richest | 0.90 | 0.73–1.11 | 0.326 |
| **Place of residence** |  |  |  |
| Rural | 1.00 | Reference | — |
| Urban | 1.06 | 0.94–1.20 | 0.346 |
| **Administrative division** |  |  |  |
| Barishal | 1.00 | Reference | — |
| Chattogram | 0.88 | 0.69–1.12 | 0.295 |
| Dhaka | 0.79 | 0.61–1.01 | 0.060 |
| Khulna | 0.83 | 0.63–1.09 | 0.178 |
| Mymensingh | 0.85 | 0.62–1.17 | 0.315 |
| Rajshahi | 0.70 | 0.53–0.92 | 0.010 |
| Rangpur | 0.96 | 0.73–1.26 | 0.758 |
| Sylhet | 0.81 | 0.61–1.08 | 0.149 |
