# Supplementary material for: Beyond conventional indicators: Reproductive coercion and its overlap with measured intimate partner violence in Bangladesh
Source: PLoS One. 2026 Oct 21;21(10):e0360372. doi: 10.1371/journal.pone.0360372 (PMC13686496; doi:10.1371/journal.pone.0360372)
Supplement: Supplementary file 3 [file pone.0360372.s003.docx]

Supplementary Table 3. Association between experience of any emotional violence within the past 12 months and patterns of reproductive coercion among currently married women in Bangladesh, 2024

| **Characteristic** | **aPR** | **95% CI** | **p-value** |
| --- | --- | --- | --- |
| **Reproductive coercion pattern** |  |  |  |
| No reproductive coercion | 1.00 | Reference | — |
| Contraceptive coercion only | 2.26 | 1.98–2.58 | <0.001 |
| Pregnancy outcome coercion only | 1.76 | 1.33–2.33 | <0.001 |
| Both contraceptive and pregnancy outcome coercion | 3.45 | 2.66–4.47 | <0.001 |
| **Women’s age (years)** |  |  |  |
| 15–19 | 1.00 | Reference | — |
| 20–29 | 0.93 | 0.77–1.12 | 0.445 |
| 30–39 | 0.83 | 0.68–1.01 | 0.058 |
| ≥40 | 0.61 | 0.49–0.75 | <0.001 |
| **Child marriage (marriage before 18 years)** |  |  |  |
| No | 1.00 | Reference | — |
| Yes | 0.91 | 0.82–1.01 | 0.070 |
| **Number of living children** |  |  |  |
| None | 1.00 | Reference | — |
| 1–2 | 1.09 | 0.83–1.43 | 0.530 |
| 3–4 | 1.12 | 0.84–1.50 | 0.429 |
| ≥5 | 1.06 | 0.75–1.49 | 0.740 |
| **Women’s education** |  |  |  |
| Primary or less | 1.00 | Reference | — |
| Secondary | 0.90 | 0.81–1.01 | 0.071 |
| Higher secondary+ | 0.86 | 0.71–1.03 | 0.105 |
| **Women’s employment status** |  |  |  |
| No | 1.00 | Reference | — |
| Yes | 0.93 | 0.67–1.29 | 0.658 |
| **Religion** |  |  |  |
| Muslim | 1.00 | Reference | — |
| Non-Muslim | 1.16 | 0.99–1.36 | 0.069 |
| **Disability status** |  |  |  |
| None | 1.00 | Reference | — |
| Some difficulty | 1.21 | 1.10–1.33 | <0.001 |
| Moderate/severe difficulty | 1.28 | 1.08–1.50 | 0.003 |
| **Husband’s education** |  |  |  |
| Primary or less | 1.00 | Reference | — |
| Secondary | 0.94 | 0.86–1.03 | 0.186 |
| Higher secondary+ | 0.79 | 0.67–0.93 | 0.005 |
| **Husband extra-marital relationship** |  |  |  |
| No | 1.00 | Reference | — |
| Yes | 2.02 | 1.81–2.26 | <0.001 |
| **Household type** |  |  |  |
| Non-slum | 1.00 | Reference | — |
| Slum | 1.10 | 0.84–1.44 | 0.488 |
| **Wealth quintile** |  |  |  |
| Poorest | 1.00 | Reference | — |
| Poorer | 0.89 | 0.80–0.99 | 0.031 |
| Middle | 0.90 | 0.77–1.04 | 0.157 |
| Richer | 0.87 | 0.74–1.01 | 0.063 |
| Richest | 0.94 | 0.79–1.12 | 0.483 |
| **Place of residence** |  |  |  |
| Rural | 1.00 | Reference | — |
| Urban | 1.09 | 0.99–1.20 | 0.076 |
| **Administrative division** |  |  |  |
| Barishal | 1.00 | Reference | — |
| Chattogram | 0.84 | 0.69–1.01 | 0.067 |
| Dhaka | 0.76 | 0.62–0.92 | 0.005 |
| Khulna | 0.80 | 0.64–0.99 | 0.041 |
| Mymensingh | 0.81 | 0.63–1.05 | 0.106 |
| Rajshahi | 0.71 | 0.57–0.88 | 0.002 |
| Rangpur | 0.97 | 0.77–1.21 | 0.770 |
| Sylhet | 0.77 | 0.61–0.97 | 0.027 |
