# Supplementary material for: Beyond conventional indicators: Reproductive coercion and its overlap with measured intimate partner violence in Bangladesh
Source: PLoS One. 2026 Oct 21;21(10):e0360372. doi: 10.1371/journal.pone.0360372 (PMC13686496; doi:10.1371/journal.pone.0360372)
Supplement: Supplementary file 4 [file pone.0360372.s004.docx]

Supplementary Table 4. Association between experience of any sexual violence within the past 12 months and patterns of reproductive coercion among currently married women in Bangladesh, 2024

| **Characteristic** | **aPR** | **95% CI** | **p-value** |
| --- | --- | --- | --- |
| **Reproductive coercion pattern** |  |  |  |
| No reproductive coercion | 1.00 | Reference | — |
| Contraceptive coercion only | 3.16 | 2.62–3.82 | <0.001 |
| Pregnancy outcome coercion only | 2.51 | 1.79–3.52 | <0.001 |
| Both contraceptive and pregnancy outcome coercion | 4.85 | 3.45–6.83 | <0.001 |
| **Women’s age (years)** |  |  |  |
| 15–19 | 1.00 | Reference | — |
| 20–29 | 0.90 | 0.69–1.18 | 0.443 |
| 30–39 | 0.78 | 0.58–1.04 | 0.092 |
| ≥40 | 0.52 | 0.38–0.71 | <0.001 |
| **Child marriage (marriage before 18 years)** |  |  |  |
| No | 1.00 | Reference | — |
| Yes | 0.95 | 0.81–1.12 | 0.545 |
| **Number of living children** |  |  |  |
| None | 1.00 | Reference | — |
| 1–2 | 1.13 | 0.74–1.72 | 0.583 |
| 3–4 | 1.18 | 0.76–1.84 | 0.459 |
| ≥5 | 1.11 | 0.66–1.87 | 0.691 |
| **Women’s education** |  |  |  |
| Primary or less | 1.00 | Reference | — |
| Secondary | 0.92 | 0.79–1.08 | 0.310 |
| Higher secondary+ | 0.84 | 0.66–1.08 | 0.170 |
| **Women’s employment status** |  |  |  |
| No | 1.00 | Reference | — |
| Yes | 0.89 | 0.58–1.37 | 0.603 |
| **Religion** |  |  |  |
| Muslim | 1.00 | Reference | — |
| Non-Muslim | 1.09 | 0.84–1.42 | 0.508 |
| **Disability status** |  |  |  |
| None | 1.00 | Reference | — |
| Some difficulty | 1.18 | 1.01–1.37 | 0.032 |
| Moderate/severe difficulty | 1.23 | 0.98–1.55 | 0.076 |
| **Husband’s education** |  |  |  |
| Primary or less | 1.00 | Reference | — |
| Secondary | 0.97 | 0.86–1.11 | 0.682 |
| Higher secondary+ | 0.81 | 0.65–1.01 | 0.062 |
| **Husband extra-marital relationship** |  |  |  |
| No | 1.00 | Reference | — |
| Yes | 2.34 | 2.00–2.74 | <0.001 |
| **Household type** |  |  |  |
| Non-slum | 1.00 | Reference | — |
| Slum | 1.12 | 0.77–1.62 | 0.547 |
| **Wealth quintile** |  |  |  |
| Poorest | 1.00 | Reference | — |
| Poorer | 0.93 | 0.80–1.09 | 0.381 |
| Middle | 0.94 | 0.77–1.15 | 0.549 |
| Richer | 0.89 | 0.72–1.10 | 0.277 |
| Richest | 0.92 | 0.73–1.16 | 0.489 |
| **Place of residence** |  |  |  |
| Rural | 1.00 | Reference | — |
| Urban | 1.04 | 0.91–1.20 | 0.542 |
| **Administrative division** |  |  |  |
| Barishal | 1.00 | Reference | — |
| Chattogram | 0.89 | 0.68–1.17 | 0.399 |
| Dhaka | 0.80 | 0.61–1.05 | 0.109 |
| Khulna | 0.82 | 0.61–1.10 | 0.181 |
| Mymensingh | 0.84 | 0.59–1.19 | 0.323 |
| Rajshahi | 0.72 | 0.53–0.98 | 0.036 |
| Rangpur | 0.95 | 0.70–1.28 | 0.729 |
| Sylhet | 0.79 | 0.58–1.08 | 0.140 |
