# Supplementary material for: Beyond conventional indicators: Reproductive coercion and its overlap with measured intimate partner violence in Bangladesh
Source: PLoS One. 2026 Oct 21;21(10):e0360372. doi: 10.1371/journal.pone.0360372 (PMC13686496; doi:10.1371/journal.pone.0360372)
Supplement: Supplementary file 5 [file pone.0360372.s005.docx]

Supplementary Table 5. Association between experience of any conventional IPV within the past 12 months and pattern of contraceptive coercion among currently married women in Bangladesh, 2024

| **Characteristic** | **aPR** | **95% CI** | **p-value** |
| --- | --- | --- | --- |
| **Contraceptive coercion typology** |  |  |  |
| No contraceptive coercion | 1.00 | Reference | — |
| Forced contraceptive use only | 2.06 | 1.84–2.31 | <0.001 |
| Prevented contraceptive use only | 2.13 | 1.78–2.55 | <0.001 |
| Both forced and prevented use | 2.51 | 1.89–3.34 | <0.001 |
| **Women’s age (years)** |  |  |  |
| 15–19 | 1.00 | Reference | — |
| 20–29 | 0.91 | 0.76–1.08 | 0.277 |
| 30–39 | 0.81 | 0.67–0.97 | 0.020 |
| ≥40 | 0.57 | 0.47–0.68 | <0.001 |
| **Child marriage (marriage before 18 years)** |  |  |  |
| No | 1.00 | Reference | — |
| Yes | 0.92 | 0.84–1.00 | 0.064 |
| **Number of living children** |  |  |  |
| None | 1.00 | Reference | — |
| 1–2 | 1.07 | 0.84–1.36 | 0.596 |
| 3–4 | 1.12 | 0.88–1.43 | 0.369 |
| ≥5 | 1.06 | 0.79–1.44 | 0.690 |
| **Women’s education** |  |  |  |
| Primary or less | 1.00 | Reference | — |
| Secondary | 0.91 | 0.82–1.00 | 0.058 |
| Higher secondary+ | 0.87 | 0.74–1.02 | 0.083 |
| **Women’s employment status** |  |  |  |
| No | 1.00 | Reference | — |
| Yes | 0.90 | 0.67–1.21 | 0.491 |
| **Religion** |  |  |  |
| Muslim | 1.00 | Reference | — |
| Non-Muslim | 1.13 | 0.99–1.30 | 0.074 |
| **Disability status** |  |  |  |
| None | 1.00 | Reference | — |
| Some difficulty | 1.20 | 1.11–1.30 | <0.001 |
| Moderate/severe difficulty | 1.26 | 1.10–1.45 | 0.001 |
| **Husband’s education** |  |  |  |
| Primary or less | 1.00 | Reference | — |
| Secondary | 0.95 | 0.88–1.02 | 0.160 |
| Higher secondary+ | 0.78 | 0.68–0.89 | <0.001 |
| **Husband extra-marital relationship** |  |  |  |
| No | 1.00 | Reference | — |
| Yes | 2.08 | 1.89–2.29 | <0.001 |
| **Household type** |  |  |  |
| Non-slum | 1.00 | Reference | — |
| Slum | 1.12 | 0.89–1.42 | 0.339 |
| **Wealth quintile** |  |  |  |
| Poorest | 1.00 | Reference | — |
| Poorer | 0.90 | 0.82–0.98 | 0.020 |
| Middle | 0.91 | 0.81–1.04 | 0.162 |
| Richer | 0.87 | 0.77–0.99 | 0.032 |
| Richest | 0.96 | 0.83–1.10 | 0.532 |
| **Place of residence** |  |  |  |
| Rural | 1.00 | Reference | — |
| Urban | 1.08 | 0.99–1.17 | 0.093 |
| **Administrative division** |  |  |  |
| Barishal | 1.00 | Reference | — |
| Chattogram | 0.85 | 0.73–0.99 | 0.041 |
| Dhaka | 0.77 | 0.66–0.89 | 0.001 |
| Khulna | 0.82 | 0.68–0.98 | 0.031 |
| Mymensingh | 0.82 | 0.66–1.01 | 0.064 |
| Rajshahi | 0.72 | 0.60–0.86 | <0.001 |
| Rangpur | 0.97 | 0.82–1.16 | 0.769 |
| Sylhet | 0.78 | 0.65–0.94 | 0.007 |
