# Supplementary material for: Beyond conventional indicators: Reproductive coercion and its overlap with measured intimate partner violence in Bangladesh
Source: PLoS One. 2026 Oct 21;21(10):e0360372. doi: 10.1371/journal.pone.0360372 (PMC13686496; doi:10.1371/journal.pone.0360372)
Supplement: Supplementary file 6 [file pone.0360372.s006.docx]

Supplementary Table 6. Association between experience of any physical violence within the past 12 months and pattern of contraceptive coercion among currently married women in Bangladesh, 2024

| **Characteristic** | **aPR** | **95% CI** | **p-value** |
| --- | --- | --- | --- |
| **Contraceptive coercion typology** |  |  |  |
| No contraceptive coercion | 1.00 | Reference | — |
| Forced contraceptive use only | 2.01 | 1.63–2.46 | <0.001 |
| Prevented contraceptive use only | 2.66 | 1.98–3.57 | <0.001 |
| Both forced and prevented use | 4.19 | 2.60–6.75 | <0.001 |
| **Women’s age (years)** |  |  |  |
| 15–19 | 1.00 | Reference | — |
| 20–29 | 0.73 | 0.57–0.94 | 0.013 |
| 30–39 | 0.59 | 0.45–0.77 | <0.001 |
| ≥40 | 0.34 | 0.26–0.45 | <0.001 |
| **Child marriage (marriage before 18 years)** |  |  |  |
| No | 1.00 | Reference | — |
| Yes | 0.90 | 0.78–1.04 | 0.140 |
| **Number of living children** |  |  |  |
| None | 1.00 | Reference | — |
| 1–2 | 1.17 | 0.81–1.69 | 0.391 |
| 3–4 | 1.16 | 0.78–1.72 | 0.463 |
| ≥5 | 1.19 | 0.73–1.94 | 0.491 |
| **Women’s education** |  |  |  |
| Primary or less | 1.00 | Reference | — |
| Secondary | 0.89 | 0.77–1.03 | 0.115 |
| Higher secondary+ | 0.66 | 0.50–0.88 | 0.005 |
| **Women’s employment status** |  |  |  |
| No | 1.00 | Reference | — |
| Yes | 0.58 | 0.33–1.01 | 0.052 |
| **Religion** |  |  |  |
| Muslim | 1.00 | Reference | — |
| Non-Muslim | 1.23 | 1.01–1.50 | 0.038 |
| **Disability status** |  |  |  |
| None | 1.00 | Reference | — |
| Some difficulty | 1.10 | 0.97–1.25 | 0.145 |
| Moderate/severe difficulty | 0.96 | 0.76–1.21 | 0.747 |
| **Husband’s education** |  |  |  |
| Primary or less | 1.00 | Reference | — |
| Secondary | 0.88 | 0.77–1.00 | 0.052 |
| Higher secondary+ | 0.68 | 0.55–0.85 | 0.001 |
| **Husband extra-marital relationship** |  |  |  |
| No | 1.00 | Reference | — |
| Yes | 3.08 | 2.68–3.52 | <0.001 |
| **Household type** |  |  |  |
| Non-slum | 1.00 | Reference | — |
| Slum | 1.49 | 1.17–1.90 | 0.001 |
| **Wealth quintile** |  |  |  |
| Poorest | 1.00 | Reference | — |
| Poorer | 0.89 | 0.77–1.02 | 0.105 |
| Middle | 0.78 | 0.67–0.92 | 0.003 |
| Richer | 0.76 | 0.62–0.92 | 0.005 |
| Richest | 0.76 | 0.61–0.96 | 0.019 |
| **Place of residence** |  |  |  |
| Rural | 1.00 | Reference | — |
| Urban | 1.14 | 1.01–1.30 | 0.042 |
| **Administrative division** |  |  |  |
| Barishal | 1.00 | Reference | — |
| Chattogram | 0.90 | 0.73–1.10 | 0.303 |
| Dhaka | 0.83 | 0.68–1.02 | 0.075 |
| Khulna | 0.88 | 0.69–1.12 | 0.297 |
| Mymensingh | 0.73 | 0.57–0.94 | 0.015 |
| Rajshahi | 0.86 | 0.69–1.09 | 0.217 |
| Rangpur | 1.22 | 1.00–1.50 | 0.053 |
| Sylhet | 0.82 | 0.60–1.11 | 0.201 |
