# Supplementary material for: Beyond conventional indicators: Reproductive coercion and its overlap with measured intimate partner violence in Bangladesh
Source: PLoS One. 2026 Oct 21;21(10):e0360372. doi: 10.1371/journal.pone.0360372 (PMC13686496; doi:10.1371/journal.pone.0360372)
Supplement: Supplementary file 7 [file pone.0360372.s007.docx]

Supplementary Table 7. Association between experience of any emotional violence within the past 12 months and pattern of contraceptive coercion among currently married women in Bangladesh, 2024

| **Characteristic** | **aPR** | **95% CI** | **p-value** |
| --- | --- | --- | --- |
| **Contraceptive coercion typology** |  |  |  |
| No contraceptive coercion | 1.00 | Reference | — |
| Forced contraceptive use only | 2.23 | 1.91–2.60 | <0.001 |
| Prevented contraceptive use only | 2.55 | 2.04–3.17 | <0.001 |
| Both forced and prevented use | 2.98 | 2.17–4.11 | <0.001 |
| **Women’s age (years)** |  |  |  |
| 15–19 | 1.00 | Reference | — |
| 20–29 | 0.88 | 0.72–1.07 | 0.191 |
| 30–39 | 0.78 | 0.63–0.96 | 0.018 |
| ≥40 | 0.60 | 0.47–0.77 | <0.001 |
| **Child marriage (marriage before 18 years)** |  |  |  |
| No | 1.00 | Reference | — |
| Yes | 0.90 | 0.81–1.01 | 0.073 |
| **Number of living children** |  |  |  |
| None | 1.00 | Reference | — |
| 1–2 | 1.05 | 0.79–1.40 | 0.729 |
| 3–4 | 1.09 | 0.81–1.46 | 0.564 |
| ≥5 | 1.03 | 0.72–1.48 | 0.875 |
| **Women’s education** |  |  |  |
| Primary or less | 1.00 | Reference | — |
| Secondary | 0.92 | 0.82–1.03 | 0.150 |
| Higher secondary+ | 0.82 | 0.67–1.00 | 0.053 |
| **Women’s employment status** |  |  |  |
| No | 1.00 | Reference | — |
| Yes | 0.79 | 0.56–1.11 | 0.174 |
| **Religion** |  |  |  |
| Muslim | 1.00 | Reference | — |
| Non-Muslim | 1.14 | 0.96–1.34 | 0.124 |
| **Disability status** |  |  |  |
| None | 1.00 | Reference | — |
| Some difficulty | 1.24 | 1.13–1.36 | <0.001 |
| Moderate/severe difficulty | 1.47 | 1.27–1.70 | <0.001 |
| **Husband’s education** |  |  |  |
| Primary or less | 1.00 | Reference | — |
| Secondary | 0.89 | 0.81–0.99 | 0.030 |
| Higher secondary+ | 0.71 | 0.60–0.85 | <0.001 |
| **Husband extra-marital relationship** |  |  |  |
| No | 1.00 | Reference | — |
| Yes | 2.61 | 2.30–2.95 | <0.001 |
| **Household type** |  |  |  |
| Non-slum | 1.00 | Reference | — |
| Slum | 1.15 | 0.88–1.51 | 0.306 |
| **Wealth quintile** |  |  |  |
| Poorest | 1.00 | Reference | — |
| Poorer | 0.91 | 0.81–1.02 | 0.113 |
| Middle | 0.86 | 0.74–1.01 | 0.060 |
| Richer | 0.85 | 0.72–1.00 | 0.049 |
| Richest | 0.87 | 0.72–1.05 | 0.147 |
| **Place of residence** |  |  |  |
| Rural | 1.00 | Reference | — |
| Urban | 1.10 | 0.99–1.22 | 0.073 |
| **Administrative division** |  |  |  |
| Barishal | 1.00 | Reference | — |
| Chattogram | 0.75 | 0.63–0.90 | 0.002 |
| Dhaka | 0.73 | 0.58–0.90 | 0.004 |
| Khulna | 0.78 | 0.64–0.96 | 0.016 |
| Mymensingh | 0.83 | 0.65–1.06 | 0.133 |
| Rajshahi | 0.70 | 0.58–0.86 | <0.001 |
| Rangpur | 0.95 | 0.77–1.18 | 0.648 |
| Sylhet | 0.75 | 0.61–0.94 | 0.011 |
