# Supplementary material for: Beyond conventional indicators: Reproductive coercion and its overlap with measured intimate partner violence in Bangladesh
Source: PLoS One. 2026 Oct 21;21(10):e0360372. doi: 10.1371/journal.pone.0360372 (PMC13686496; doi:10.1371/journal.pone.0360372)
Supplement: Supplementary file 8 [file pone.0360372.s008.docx]

Supplementary Table 8. Association between experience of any sexual violence within the past 12 months and pattern of contraceptive coercion among currently married women in Bangladesh, 2024

| **Characteristic** | **aPR** | **95% CI** | **p-value** |
| --- | --- | --- | --- |
| **Contraceptive coercion typology** |  |  |  |
| No contraceptive coercion | 1.00 | Reference | — |
| Forced contraceptive use only | 3.24 | 2.62–4.00 | <0.001 |
| Prevented contraceptive use only | 3.00 | 2.17–4.15 | <0.001 |
| Both forced and prevented use | 4.41 | 2.70–7.19 | <0.001 |
| **Women’s age (years)** |  |  |  |
| 15–19 | 1.00 | Reference | — |
| 20–29 | 0.79 | 0.60–1.03 | 0.085 |
| 30–39 | 0.67 | 0.50–0.91 | 0.010 |
| ≥40 | 0.42 | 0.30–0.58 | <0.001 |
| **Child marriage (marriage before 18 years)** |  |  |  |
| No | 1.00 | Reference | — |
| Yes | 0.94 | 0.79–1.13 | 0.505 |
| **Number of living children** |  |  |  |
| None | 1.00 | Reference | — |
| 1–2 | 1.10 | 0.69–1.74 | 0.695 |
| 3–4 | 1.15 | 0.72–1.84 | 0.554 |
| ≥5 | 1.06 | 0.61–1.85 | 0.827 |
| **Women’s education** |  |  |  |
| Primary or less | 1.00 | Reference | — |
| Secondary | 0.82 | 0.69–0.98 | 0.030 |
| Higher secondary+ | 0.77 | 0.59–1.00 | 0.052 |
| **Women’s employment status** |  |  |  |
| No | 1.00 | Reference | — |
| Yes | 0.72 | 0.46–1.12 | 0.144 |
| **Religion** |  |  |  |
| Muslim | 1.00 | Reference | — |
| Non-Muslim | 1.05 | 0.78–1.40 | 0.759 |
| **Disability status** |  |  |  |
| None | 1.00 | Reference | — |
| Some difficulty | 1.23 | 1.04–1.47 | 0.018 |
| Moderate/severe difficulty | 1.19 | 0.92–1.54 | 0.187 |
| **Husband’s education** |  |  |  |
| Primary or less | 1.00 | Reference | — |
| Secondary | 0.89 | 0.77–1.04 | 0.138 |
| Higher secondary+ | 0.74 | 0.59–0.93 | 0.012 |
| **Husband extra-marital relationship** |  |  |  |
| No | 1.00 | Reference | — |
| Yes | 1.96 | 1.60–2.40 | <0.001 |
| **Household type** |  |  |  |
| Non-slum | 1.00 | Reference | — |
| Slum | 1.18 | 0.84–1.66 | 0.336 |
| **Wealth quintile** |  |  |  |
| Poorest | 1.00 | Reference | — |
| Poorer | 1.05 | 0.85–1.30 | 0.658 |
| Middle | 1.09 | 0.85–1.40 | 0.502 |
| Richer | 1.12 | 0.86–1.45 | 0.393 |
| Richest | 1.29 | 1.02–1.64 | 0.034 |
| **Place of residence** |  |  |  |
| Rural | 1.00 | Reference | — |
| Urban | 1.14 | 1.01–1.30 | 0.041 |
| **Administrative division** |  |  |  |
| Barishal | 1.00 | Reference | — |
| Chattogram | 0.82 | 0.61–1.10 | 0.189 |
| Dhaka | 0.67 | 0.54–0.83 | <0.001 |
| Khulna | 0.69 | 0.51–0.94 | 0.017 |
| Mymensingh | 0.63 | 0.47–0.85 | 0.002 |
| Rajshahi | 0.67 | 0.50–0.90 | 0.007 |
| Rangpur | 0.76 | 0.58–0.99 | 0.038 |
| Sylhet | 0.81 | 0.59–1.10 | 0.177 |
