# Supplementary material for: Beyond conventional indicators: Reproductive coercion and its overlap with measured intimate partner violence in Bangladesh
Source: PLoS One. 2026 Oct 21;21(10):e0360372. doi: 10.1371/journal.pone.0360372 (PMC13686496; doi:10.1371/journal.pone.0360372)
Supplement: Supplementary file 9 [file pone.0360372.s009.docx]

Supplementary Table 9. Association between experience of any conventional IPV over the lifetime and patterns of reproductive coercion among ever-married women in Bangladesh, 2024

| **Characteristic** | **aPR** | **95% CI** | **p-value** |
| --- | --- | --- | --- |
| **Reproductive coercion pattern** |  |  |  |
| No reproductive coercion | 1.00 | Reference | — |
| Contraceptive coercion only | 1.36 | 1.29–1.43 | <0.001 |
| Pregnancy outcome coercion only | 1.58 | 1.53–1.64 | <0.001 |
| Both contraceptive and pregnancy outcome coercion | 1.53 | 1.47–1.60 | <0.001 |
| **Women’s age (years)** |  |  |  |
| 15–19 | 1.00 | Reference | — |
| 20–29 | 1.16 | 0.99–1.37 | 0.070 |
| 30–39 | 1.24 | 1.06–1.46 | 0.007 |
| ≥40 | 1.23 | 1.05–1.44 | 0.012 |
| **Child marriage (marriage before 18 years)** |  |  |  |
| No | 1.00 | Reference | — |
| Yes | 1.04 | 0.99–1.09 | 0.108 |
| **Number of living children** |  |  |  |
| None | 1.00 | Reference | — |
| 1–2 | 1.05 | 0.92–1.21 | 0.439 |
| 3–4 | 1.05 | 0.91–1.20 | 0.521 |
| ≥5 | 1.05 | 0.91–1.22 | 0.492 |
| **Women’s education** |  |  |  |
| Primary or less | 1.00 | Reference | — |
| Secondary | 0.94 | 0.90–0.98 | 0.003 |
| Higher secondary+ | 0.90 | 0.81–1.00 | 0.043 |
| **Women’s employment status** |  |  |  |
| No | 1.00 | Reference | — |
| Yes | 1.00 | 0.90–1.10 | 0.975 |
| **Religion** |  |  |  |
| Muslim | 1.00 | Reference | — |
| Non-Muslim | 0.94 | 0.87–1.02 | 0.136 |
| **Disability status** |  |  |  |
| None | 1.00 | Reference | — |
| Some difficulty | 1.13 | 1.09–1.17 | <0.001 |
| Moderate/severe difficulty | 1.16 | 1.10–1.22 | <0.001 |
| **Husband’s education** |  |  |  |
| Primary or less | 1.00 | Reference | — |
| Secondary | 0.94 | 0.90–0.99 | 0.024 |
| Higher secondary+ | 0.85 | 0.78–0.91 | <0.001 |
| **Husband extra-marital relationship** |  |  |  |
| No | 1.00 | Reference | — |
| Yes | 1.45 | 1.41–1.50 | <0.001 |
| **Household type** |  |  |  |
| Non-slum | 1.00 | Reference | — |
| Slum | 1.12 | 1.02–1.24 | 0.023 |
| **Wealth quintile** |  |  |  |
| Poorest | 1.00 | Reference | — |
| Poorer | 0.98 | 0.94–1.02 | 0.332 |
| Middle | 0.95 | 0.90–1.00 | 0.049 |
| Richer | 0.93 | 0.87–0.98 | 0.015 |
| Richest | 0.91 | 0.85–0.99 | 0.021 |
| **Place of residence** |  |  |  |
| Rural | 1.00 | Reference | — |
| Urban | 1.03 | 0.99–1.08 | 0.190 |
| **Administrative division** |  |  |  |
| Barishal | 1.00 | Reference | — |
| Chattogram | 0.97 | 0.88–1.06 | 0.499 |
| Dhaka | 0.96 | 0.88–1.04 | 0.314 |
| Khulna | 1.02 | 0.93–1.13 | 0.639 |
| Mymensingh | 0.90 | 0.81–1.00 | 0.053 |
| Rajshahi | 0.97 | 0.88–1.06 | 0.484 |
| Rangpur | 0.97 | 0.88–1.07 | 0.521 |
| Sylhet | 0.81 | 0.73–0.90 | <0.001 |
