# Supplementary material for: Beyond conventional indicators: Reproductive coercion and its overlap with measured intimate partner violence in Bangladesh
Source: PLoS One. 2026 Oct 21;21(10):e0360372. doi: 10.1371/journal.pone.0360372 (PMC13686496; doi:10.1371/journal.pone.0360372)
Supplement: Supplementary file 11 [file pone.0360372.s011.docx]

Supplementary Table 11. Association between experience of any emotional violence over the lifetime and patterns of reproductive coercion among ever-married women in Bangladesh, 2024

| **Characteristic** | **aPR** | **95% CI** | **p-value** |
| --- | --- | --- | --- |
| **Reproductive coercion pattern** |  |  |  |
| No reproductive coercion | 1.00 | Reference | — |
| Contraceptive coercion only | 1.64 | 1.53–1.76 | <0.001 |
| Pregnancy outcome coercion only | 1.70 | 1.57–1.83 | <0.001 |
| Both contraceptive and pregnancy outcome coercion | 1.84 | 1.68–2.03 | <0.001 |
| **Women’s age (years)** |  |  |  |
| 15–19 | 1.00 | Reference | — |
| 20–29 | 1.24 | 1.01–1.52 | 0.044 |
| 30–39 | 1.34 | 1.08–1.65 | 0.007 |
| ≥40 | 1.29 | 1.04–1.60 | 0.021 |
| **Child marriage (marriage before 18 years)** |  |  |  |
| No | 1.00 | Reference | — |
| Yes | 1.05 | 0.98–1.12 | 0.141 |
| **Number of living children** |  |  |  |
| None | 1.00 | Reference | — |
| 1–2 | 1.03 | 0.83–1.27 | 0.796 |
| 3–4 | 1.01 | 0.81–1.26 | 0.944 |
| ≥5 | 0.99 | 0.79–1.25 | 0.943 |
| **Women’s education** |  |  |  |
| Primary or less | 1.00 | Reference | — |
| Secondary | 0.92 | 0.86–0.98 | 0.006 |
| Higher secondary+ | 0.83 | 0.72–0.96 | 0.012 |
| **Women’s employment status** |  |  |  |
| No | 1.00 | Reference | — |
| Yes | 1.01 | 0.89–1.14 | 0.920 |
| **Religion** |  |  |  |
| Muslim | 1.00 | Reference | — |
| Non-Muslim | 0.95 | 0.86–1.04 | 0.247 |
| **Disability status** |  |  |  |
| None | 1.00 | Reference | — |
| Some difficulty | 1.20 | 1.13–1.27 | <0.001 |
| Moderate/severe difficulty | 1.40 | 1.30–1.52 | <0.001 |
| **Husband’s education** |  |  |  |
| Primary or less | 1.00 | Reference | — |
| Secondary | 0.90 | 0.82–0.98 | 0.022 |
| Higher secondary+ | 0.77 | 0.68–0.86 | <0.001 |
| **Husband extra-marital relationship** |  |  |  |
| No | 1.00 | Reference | — |
| Yes | 2.25 | 2.15–2.36 | <0.001 |
| **Household type** |  |  |  |
| Non-slum | 1.00 | Reference | — |
| Slum | 1.15 | 1.01–1.32 | 0.041 |
| **Wealth quintile** |  |  |  |
| Poorest | 1.00 | Reference | — |
| Poorer | 0.95 | 0.89–1.01 | 0.117 |
| Middle | 0.89 | 0.82–0.96 | 0.005 |
| Richer | 0.86 | 0.79–0.95 | 0.002 |
| Richest | 0.88 | 0.79–0.99 | 0.028 |
| **Place of residence** |  |  |  |
| Rural | 1.00 | Reference | — |
| Urban | 1.09 | 1.03–1.16 | 0.004 |
| **Administrative division** |  |  |  |
| Barishal | 1.00 | Reference | — |
| Chattogram | 0.86 | 0.75–0.98 | 0.022 |
| Dhaka | 0.87 | 0.78–0.98 | 0.022 |
| Khulna | 0.93 | 0.82–1.05 | 0.243 |
| Mymensingh | 0.82 | 0.69–0.99 | 0.036 |
| Rajshahi | 0.85 | 0.74–0.96 | 0.012 |
| Rangpur | 0.94 | 0.83–1.07 | 0.340 |
| Sylhet | 0.77 | 0.67–0.89 | 0.001 |
