# Supplementary material for: Beyond conventional indicators: Reproductive coercion and its overlap with measured intimate partner violence in Bangladesh
Source: PLoS One. 2026 Oct 21;21(10):e0360372. doi: 10.1371/journal.pone.0360372 (PMC13686496; doi:10.1371/journal.pone.0360372)
Supplement: Supplementary file 12 [file pone.0360372.s012.docx]

Supplementary Table 12. Association between experience of any sexual violence over the lifetime and patterns of reproductive coercion among ever-married women in Bangladesh, 2024

| **Characteristic** | **aPR** | **95% CI** | **p-value** |
| --- | --- | --- | --- |
| **Reproductive coercion pattern** |  |  |  |
| No reproductive coercion | 1.00 | Reference | — |
| Contraceptive coercion only | 1.96 | 1.79–2.15 | <0.001 |
| Pregnancy outcome coercion only | 3.12 | 2.94–3.31 | <0.001 |
| Both contraceptive and pregnancy outcome coercion | 2.95 | 2.74–3.17 | <0.001 |
| **Women’s age (years)** |  |  |  |
| 15–19 | 1.00 | Reference | — |
| 20–29 | 1.10 | 0.89–1.37 | 0.376 |
| 30–39 | 1.15 | 0.92–1.43 | 0.220 |
| ≥40 | 1.09 | 0.87–1.37 | 0.456 |
| **Child marriage (marriage before 18 years)** |  |  |  |
| No | 1.00 | Reference | — |
| Yes | 1.03 | 0.96–1.10 | 0.432 |
| **Number of living children** |  |  |  |
| None | 1.00 | Reference | — |
| 1–2 | 1.02 | 0.82–1.27 | 0.874 |
| 3–4 | 1.01 | 0.80–1.27 | 0.947 |
| ≥5 | 1.04 | 0.82–1.32 | 0.726 |
| **Women’s education** |  |  |  |
| Primary or less | 1.00 | Reference | — |
| Secondary | 0.89 | 0.83–0.96 | 0.003 |
| Higher secondary+ | 0.85 | 0.73–0.98 | 0.028 |
| **Women’s employment status** |  |  |  |
| No | 1.00 | Reference | — |
| Yes | 1.04 | 0.91–1.18 | 0.568 |
| **Religion** |  |  |  |
| Muslim | 1.00 | Reference | — |
| Non-Muslim | 0.96 | 0.86–1.07 | 0.441 |
| **Disability status** |  |  |  |
| None | 1.00 | Reference | — |
| Some difficulty | 1.17 | 1.09–1.26 | <0.001 |
| Moderate/severe difficulty | 1.23 | 1.11–1.36 | <0.001 |
| **Husband’s education** |  |  |  |
| Primary or less | 1.00 | Reference | — |
| Secondary | 0.93 | 0.87–0.99 | 0.024 |
| Higher secondary+ | 0.79 | 0.70–0.89 | <0.001 |
| **Husband extra-marital relationship** |  |  |  |
| No | 1.00 | Reference | — |
| Yes | 1.72 | 1.56–1.90 | <0.001 |
| **Household type** |  |  |  |
| Non-slum | 1.00 | Reference | — |
| Slum | 1.08 | 0.93–1.25 | 0.322 |
| **Wealth quintile** |  |  |  |
| Poorest | 1.00 | Reference | — |
| Poorer | 0.97 | 0.90–1.05 | 0.475 |
| Middle | 0.95 | 0.87–1.04 | 0.254 |
| Richer | 0.96 | 0.87–1.06 | 0.421 |
| Richest | 1.01 | 0.91–1.13 | 0.804 |
| **Place of residence** |  |  |  |
| Rural | 1.00 | Reference | — |
| Urban | 1.11 | 1.03–1.18 | 0.004 |
| **Administrative division** |  |  |  |
| Barishal | 1.00 | Reference | — |
| Chattogram | 0.94 | 0.82–1.08 | 0.384 |
| Dhaka | 0.92 | 0.82–1.03 | 0.150 |
| Khulna | 0.97 | 0.84–1.12 | 0.676 |
| Mymensingh | 0.69 | 0.58–0.83 | <0.001 |
| Rajshahi | 0.93 | 0.81–1.07 | 0.306 |
| Rangpur | 0.95 | 0.83–1.10 | 0.503 |
| Sylhet | 0.88 | 0.76–1.02 | 0.086 |
