# Supplementary material for: Beyond conventional indicators: Reproductive coercion and its overlap with measured intimate partner violence in Bangladesh
Source: PLoS One. 2026 Oct 21;21(10):e0360372. doi: 10.1371/journal.pone.0360372 (PMC13686496; doi:10.1371/journal.pone.0360372)
Supplement: Supplementary file 13 [file pone.0360372.s013.docx]

Supplementary Table 13. Association between experience of any conventional IPV over the lifetime and patterns of contraception coercion among ever-married women in Bangladesh, 2024

| **Characteristic** | **aPR** | **95% CI** | **p-value** |
| --- | --- | --- | --- |
| **Contraceptive coercion typology** |  |  |  |
| No contraceptive coercion | 1.00 | Reference | — |
| Forced contraceptive use only | 1.32 | 1.25–1.40 | <0.001 |
| Prevented contraceptive use only | 1.41 | 1.33–1.49 | <0.001 |
| Both forced and prevented use | 1.41 | 1.31–1.52 | <0.001 |
| **Women’s age (years)** |  |  |  |
| 15–19 | 1.00 | Reference | — |
| 20–29 | 1.17 | 0.99–1.38 | 0.071 |
| 30–39 | 1.25 | 1.06–1.46 | 0.007 |
| ≥40 | 1.22 | 1.03–1.43 | 0.019 |
| **Child marriage (marriage before 18 years)** |  |  |  |
| No | 1.00 | Reference | — |
| Yes | 1.04 | 0.99–1.09 | 0.082 |
| **Number of living children** |  |  |  |
| None | 1.00 | Reference | — |
| 1–2 | 1.02 | 0.89–1.17 | 0.734 |
| 3–4 | 1.02 | 0.89–1.18 | 0.750 |
| ≥5 | 1.03 | 0.88–1.19 | 0.743 |
| **Women’s education** |  |  |  |
| Primary or less | 1.00 | Reference | — |
| Secondary | 0.94 | 0.90–0.98 | 0.004 |
| Higher secondary+ | 0.90 | 0.81–1.00 | 0.046 |
| **Women’s employment status** |  |  |  |
| No | 1.00 | Reference | — |
| Yes | 1.01 | 0.91–1.12 | 0.900 |
| **Religion** |  |  |  |
| Muslim | 1.00 | Reference | — |
| Non-Muslim | 0.94 | 0.86–1.01 | 0.099 |
| **Disability status** |  |  |  |
| None | 1.00 | Reference | — |
| Some difficulty | 1.14 | 1.10–1.18 | <0.001 |
| Moderate/severe difficulty | 1.18 | 1.12–1.24 | <0.001 |
| **Husband’s education** |  |  |  |
| Primary or less | 1.00 | Reference | — |
| Secondary | 0.94 | 0.89–0.99 | 0.023 |
| Higher secondary+ | 0.84 | 0.78–0.91 | <0.001 |
| **Husband extra-marital relationship** |  |  |  |
| No | 1.00 | Reference | — |
| Yes | 1.49 | 1.44–1.54 | <0.001 |
| **Household type** |  |  |  |
| Non-slum | 1.00 | Reference | — |
| Slum | 1.14 | 1.03–1.25 | 0.008 |
| **Wealth quintile** |  |  |  |
| Poorest | 1.00 | Reference | — |
| Poorer | 0.98 | 0.94–1.02 | 0.266 |
| Middle | 0.95 | 0.90–1.00 | 0.060 |
| Richer | 0.93 | 0.87–0.99 | 0.019 |
| Richest | 0.93 | 0.86–1.00 | 0.060 |
| **Place of residence** |  |  |  |
| Rural | 1.00 | Reference | — |
| Urban | 1.03 | 0.98–1.07 | 0.262 |
| **Administrative division** |  |  |  |
| Barishal | 1.00 | Reference | — |
| Chattogram | 0.97 | 0.89–1.07 | 0.580 |
| Dhaka | 0.95 | 0.87–1.03 | 0.214 |
| Khulna | 1.02 | 0.93–1.13 | 0.636 |
| Mymensingh | 0.89 | 0.80–0.99 | 0.026 |
| Rajshahi | 0.96 | 0.88–1.05 | 0.395 |
| Rangpur | 0.97 | 0.88–1.07 | 0.565 |
| Sylhet | 0.81 | 0.73–0.90 | <0.001 |
