# Supplementary material for: Beyond conventional indicators: Reproductive coercion and its overlap with measured intimate partner violence in Bangladesh
Source: PLoS One. 2026 Oct 21;21(10):e0360372. doi: 10.1371/journal.pone.0360372 (PMC13686496; doi:10.1371/journal.pone.0360372)
Supplement: Supplementary file 14 [file pone.0360372.s014.docx]

Supplementary Table 14. Association between experience of any physical violence over the lifetime and patterns of contraception coercion among ever-married women in Bangladesh, 2024

| **Characteristic** | **aPR** | **95% CI** | **p-value** |
| --- | --- | --- | --- |
| **Contraceptive coercion typology** |  |  |  |
| No contraceptive coercion | 1.00 | Reference | — |
| Forced contraceptive use only | 1.33 | 1.22–1.44 | <0.001 |
| Prevented contraceptive use only | 1.44 | 1.31–1.58 | <0.001 |
| Both forced and prevented use | 1.41 | 1.25–1.60 | <0.001 |
| **Women’s age (years)** |  |  |  |
| 15–19 | 1.00 | Reference | — |
| 20–29 | 1.20 | 0.96–1.48 | 0.103 |
| 30–39 | 1.30 | 1.06–1.59 | 0.011 |
| ≥40 | 1.30 | 1.05–1.61 | 0.014 |
| **Child marriage (marriage before 18 years)** |  |  |  |
| No | 1.00 | Reference | — |
| Yes | 1.07 | 1.00–1.14 | 0.038 |
| **Number of living children** |  |  |  |
| None | 1.00 | Reference | — |
| 1–2 | 1.04 | 0.87–1.26 | 0.651 |
| 3–4 | 1.03 | 0.84–1.25 | 0.793 |
| ≥5 | 1.05 | 0.86–1.29 | 0.611 |
| **Women’s education** |  |  |  |
| Primary or less | 1.00 | Reference | — |
| Secondary | 0.93 | 0.88–0.98 | 0.003 |
| Higher secondary+ | 0.78 | 0.66–0.90 | 0.001 |
| **Women’s employment status** |  |  |  |
| No | 1.00 | Reference | — |
| Yes | 1.05 | 0.93–1.18 | 0.420 |
| **Religion** |  |  |  |
| Muslim | 1.00 | Reference | — |
| Non-Muslim | 0.92 | 0.84–1.01 | 0.073 |
| **Disability status** |  |  |  |
| None | 1.00 | Reference | — |
| Some difficulty | 1.15 | 1.10–1.20 | <0.001 |
| Moderate/severe difficulty | 1.20 | 1.11–1.29 | <0.001 |
| **Husband’s education** |  |  |  |
| Primary or less | 1.00 | Reference | — |
| Secondary | 0.92 | 0.85–0.99 | 0.020 |
| Higher secondary+ | 0.77 | 0.69–0.85 | <0.001 |
| **Husband extra-marital relationship** |  |  |  |
| No | 1.00 | Reference | — |
| Yes | 1.72 | 1.65–1.81 | <0.001 |
| **Household type** |  |  |  |
| Non-slum | 1.00 | Reference | — |
| Slum | 1.28 | 1.11–1.48 | 0.001 |
| **Wealth quintile** |  |  |  |
| Poorest | 1.00 | Reference | — |
| Poorer | 0.96 | 0.91–1.01 | 0.132 |
| Middle | 0.94 | 0.88–1.00 | 0.064 |
| Richer | 0.91 | 0.84–0.98 | 0.011 |
| Richest | 0.82 | 0.74–0.91 | <0.001 |
| **Place of residence** |  |  |  |
| Rural | 1.00 | Reference | — |
| Urban | 1.01 | 0.95–1.07 | 0.813 |
| **Administrative division** |  |  |  |
| Barishal | 1.00 | Reference | — |
| Chattogram | 0.97 | 0.88–1.08 | 0.596 |
| Dhaka | 0.98 | 0.89–1.09 | 0.732 |
| Khulna | 1.14 | 1.03–1.27 | 0.015 |
| Mymensingh | 0.88 | 0.77–0.99 | 0.033 |
| Rajshahi | 1.02 | 0.93–1.13 | 0.640 |
| Rangpur | 1.05 | 0.94–1.16 | 0.396 |
| Sylhet | 0.68 | 0.59–0.78 | <0.001 |
