# Supplementary material for: Beyond conventional indicators: Reproductive coercion and its overlap with measured intimate partner violence in Bangladesh
Source: PLoS One. 2026 Oct 21;21(10):e0360372. doi: 10.1371/journal.pone.0360372 (PMC13686496; doi:10.1371/journal.pone.0360372)
Supplement: Supplementary file 15 [file pone.0360372.s015.docx]

Supplementary Table 15. Association between experience of any emotional violence over the lifetime and patterns of contraception coercion among ever-married women in Bangladesh, 2024

| **Characteristic** | **aPR** | **95% CI** | **p-value** |
| --- | --- | --- | --- |
| **Contraceptive coercion typology** |  |  |  |
| No contraceptive coercion | 1.00 | Reference | — |
| Forced contraceptive use only | 1.58 | 1.45–1.71 | <0.001 |
| Prevented contraceptive use only | 1.69 | 1.53–1.87 | <0.001 |
| Both forced and prevented use | 1.72 | 1.47–2.00 | <0.001 |
| **Women’s age (years)** |  |  |  |
| 15–19 | 1.00 | Reference | — |
| 20–29 | 1.23 | 1.00–1.52 | 0.047 |
| 30–39 | 1.33 | 1.08–1.64 | 0.007 |
| ≥40 | 1.21 | 0.97–1.50 | 0.088 |
| **Child marriage (marriage before 18 years)** |  |  |  |
| No | 1.00 | Reference | — |
| Yes | 1.03 | 0.96–1.10 | 0.407 |
| **Number of living children** |  |  |  |
| None | 1.00 | Reference | — |
| 1–2 | 1.04 | 0.85–1.27 | 0.729 |
| 3–4 | 1.05 | 0.85–1.30 | 0.632 |
| ≥5 | 1.05 | 0.84–1.30 | 0.689 |
| **Women’s education** |  |  |  |
| Primary or less | 1.00 | Reference | — |
| Secondary | 0.92 | 0.86–0.98 | 0.007 |
| Higher secondary+ | 0.91 | 0.81–1.04 | 0.165 |
| **Women’s employment status** |  |  |  |
| No | 1.00 | Reference | — |
| Yes | 1.05 | 0.84–1.31 | 0.685 |
| **Religion** |  |  |  |
| Muslim | 1.00 | Reference | — |
| Non-Muslim | 0.96 | 0.86–1.08 | 0.520 |
| **Disability status** |  |  |  |
| None | 1.00 | Reference | — |
| Some difficulty | 1.21 | 1.15–1.28 | <0.001 |
| Moderate/severe difficulty | 1.43 | 1.32–1.55 | <0.001 |
| **Husband’s education** |  |  |  |
| Primary or less | 1.00 | Reference | — |
| Secondary | 0.90 | 0.82–0.98 | 0.021 |
| Higher secondary+ | 0.76 | 0.68–0.85 | <0.001 |
| **Husband extra-marital relationship** |  |  |  |
| No | 1.00 | Reference | — |
| Yes | 2.32 | 2.21–2.43 | <0.001 |
| **Household type** |  |  |  |
| Non-slum | 1.00 | Reference | — |
| Slum | 1.18 | 1.03–1.34 | 0.018 |
| **Wealth quintile** |  |  |  |
| Poorest | 1.00 | Reference | — |
| Poorer | 0.96 | 0.90–1.03 | 0.259 |
| Middle | 0.89 | 0.82–0.97 | 0.006 |
| Richer | 0.87 | 0.79–0.95 | 0.003 |
| Richest | 0.97 | 0.87–1.08 | 0.532 |
| **Place of residence** |  |  |  |
| Rural | 1.00 | Reference | — |
| Urban | 1.09 | 1.02–1.15 | 0.007 |
| **Administrative division** |  |  |  |
| Barishal | 1.00 | Reference | — |
| Chattogram | 0.86 | 0.76–0.98 | 0.028 |
| Dhaka | 0.86 | 0.76–0.97 | 0.012 |
| Khulna | 0.91 | 0.80–1.03 | 0.136 |
| Mymensingh | 0.81 | 0.67–0.97 | 0.021 |
| Rajshahi | 0.84 | 0.73–0.96 | 0.009 |
| Rangpur | 0.91 | 0.80–1.04 | 0.179 |
| Sylhet | 0.77 | 0.66–0.89 | 0.001 |
