# Supplementary material for: Beyond conventional indicators: Reproductive coercion and its overlap with measured intimate partner violence in Bangladesh
Source: PLoS One. 2026 Oct 21;21(10):e0360372. doi: 10.1371/journal.pone.0360372 (PMC13686496; doi:10.1371/journal.pone.0360372)
Supplement: Supplementary file 16 [file pone.0360372.s016.docx]

Supplementary Table 16. Association between experience of any sexual violence over the lifetime and patterns of contraception coercion among ever-married women in Bangladesh, 2024

| **Characteristic** | **aPR** | **95% CI** | **p-value** |
| --- | --- | --- | --- |
| **Contraceptive coercion typology** |  |  |  |
| No contraceptive coercion | 1.00 | Reference | — |
| Forced contraceptive use only | 1.95 | 1.77–2.15 | <0.001 |
| Prevented contraceptive use only | 2.02 | 1.82–2.23 | <0.001 |
| Both forced and prevented use | 1.99 | 1.70–2.32 | <0.001 |
| **Women’s age (years)** |  |  |  |
| 15–19 | 1.00 | Reference | — |
| 20–29 | 1.06 | 0.86–1.30 | 0.600 |
| 30–39 | 1.15 | 0.94–1.40 | 0.166 |
| ≥40 | 1.07 | 0.87–1.32 | 0.521 |
| **Child marriage (marriage before 18 years)** |  |  |  |
| No | 1.00 | Reference | — |
| Yes | 1.03 | 0.95–1.11 | 0.476 |
| **Number of living children** |  |  |  |
| None | 1.00 | Reference | — |
| 1–2 | 0.95 | 0.75–1.21 | 0.675 |
| 3–4 | 0.99 | 0.77–1.28 | 0.947 |
| ≥5 | 1.06 | 0.82–1.38 | 0.647 |
| **Women’s education** |  |  |  |
| Primary or less | 1.00 | Reference | — |
| Secondary | 0.89 | 0.82–0.97 | 0.005 |
| Higher secondary+ | 0.85 | 0.73–0.99 | 0.037 |
| **Women’s employment status** |  |  |  |
| No | 1.00 | Reference | — |
| Yes | 0.91 | 0.72–1.13 | 0.388 |
| **Religion** |  |  |  |
| Muslim | 1.00 | Reference | — |
| Non-Muslim | 0.90 | 0.79–1.02 | 0.106 |
| **Disability status** |  |  |  |
| None | 1.00 | Reference | — |
| Some difficulty | 1.21 | 1.13–1.30 | <0.001 |
| Moderate/severe difficulty | 1.30 | 1.17–1.44 | <0.001 |
| **Husband’s education** |  |  |  |
| Primary or less | 1.00 | Reference | — |
| Secondary | 0.92 | 0.86–0.99 | 0.021 |
| Higher secondary+ | 0.77 | 0.68–0.87 | <0.001 |
| **Husband extra-marital relationship** |  |  |  |
| No | 1.00 | Reference | — |
| Yes | 1.87 | 1.68–2.07 | <0.001 |
| **Household type** |  |  |  |
| Non-slum | 1.00 | Reference | — |
| Slum | 1.05 | 0.78–1.42 | 0.732 |
| **Wealth quintile** |  |  |  |
| Poorest | 1.00 | Reference | — |
| Poorer | 0.96 | 0.88–1.04 | 0.316 |
| Middle | 0.96 | 0.87–1.05 | 0.325 |
| Richer | 0.92 | 0.82–1.02 | 0.099 |
| Richest | 1.01 | 0.88–1.15 | 0.920 |
| **Place of residence** |  |  |  |
| Rural | 1.00 | Reference | — |
| Urban | 1.09 | 1.02–1.18 | 0.016 |
| **Administrative division** |  |  |  |
| Barishal | 1.00 | Reference | — |
| Chattogram | 1.08 | 0.93–1.25 | 0.325 |
| Dhaka | 0.85 | 0.73–0.98 | 0.030 |
| Khulna | 0.92 | 0.78–1.07 | 0.270 |
| Mymensingh | 0.65 | 0.54–0.79 | <0.001 |
| Rajshahi | 0.88 | 0.73–1.05 | 0.153 |
| Rangpur | 0.88 | 0.75–1.03 | 0.117 |
| Sylhet | 0.87 | 0.74–1.03 | 0.105 |
