# Supplementary material for: Beyond conventional indicators: Reproductive coercion and its overlap with measured intimate partner violence in Bangladesh
Source: PLoS One. 2026 Oct 21;21(10):e0360372. doi: 10.1371/journal.pone.0360372 (PMC13686496; doi:10.1371/journal.pone.0360372)
Supplement: Supplementary file 17 [file pone.0360372.s017.docx]

Supplementary Table 17. Distribution of joint exposure to conventional IPV and reproductive coercion by respondent characteristics among ever-married women in Bangladesh, 2024

|  | **Past 12 months experience** | | | | **Lifetime experience** | | | |
| --- | --- | --- | --- | --- | --- | --- | --- | --- |
| **Characteristics** | **Neither conventional violence nor reproductive coercion** | **Conventional violence only** | **Reproductive coercion only** | **Both conventional violence and reproductive coercion** | **Neither conventional violence nor reproductive coercion** | **Conventional only** | **Reproductive coercion only** | **Both conventional violence and reproductive coercion** |
| **Women’s age (years)** |  |  |  |  |  |  |  |  |
| 15–19 | 64.85 | 26.04 | 3.02 | 6.09 | 53.56 | 34.13 | 1.97 | 10.33 |
| 20–29 | 68.72 | 26.70 | 1.59 | 3.00 | 44.95 | 46.26 | 0.93 | 7.86 |
| 30–39 | 69.41 | 26.13 | 1.55 | 2.91 | 36.84 | 52.63 | 0.62 | 9.92 |
| ≥40 | 81.69 | 17.31 | 0.44 | 0.56 | 34.72 | 56.90 | 0.64 | 7.74 |
| **Child marriage (marriage before 18 years)** |  |  |  |  |  |  |  |  |
| No | 74.01 | 22.62 | 1.22 | 2.15 | 46.40 | 45.84 | 0.94 | 6.82 |
| Yes | 74.62 | 22.22 | 1.12 | 2.04 | 36.09 | 54.12 | 0.72 | 9.07 |
| **Number of living children** |  |  |  |  |  |  |  |  |
| None | 71.51 | 21.84 | 1.16 | 5.50 | 47.22 | 40.90 | 0.48 | 11.40 |
| 1–2 | 72.64 | 23.99 | 1.14 | 2.24 | 40.03 | 51.57 | 0.58 | 7.83 |
| 3–4 | 75.05 | 22.30 | 1.12 | 1.53 | 35.22 | 55.26 | 0.83 | 8.69 |
| ≥5 | 83.25 | 16.17 | 0.04 | 0.54 | 34.14 | 55.08 | 0.64 | 10.14 |
| **Women’s education** |  |  |  |  |  |  |  |  |
| Primary or less | 75.65 | 21.95 | 0.72 | 1.68 | 31.94 | 58.48 | 0.75 | 8.83 |
| Secondary | 72.13 | 23.43 | 1.67 | 2.77 | 43.36 | 47.00 | 0.80 | 8.83 |
| Higher secondary+ | 76.90 | 20.32 | 1.34 | 1.44 | 53.88 | 39.54 | 0.82 | 5.76 |
| **Women’s employment status** |  |  |  |  |  |  |  |  |
| No | 74.23 | 22.47 | 1.17 | 2.13 | 38.50 | 52.12 | 0.79 | 8.60 |
| Yes | 79.86 | 18.92 | 0.59 | 0.63 | 46.29 | 47.57 | 0.45 | 5.70 |
| **Religion** |  |  |  |  |  |  |  |  |
| Muslim | 74.27 | 22.38 | 1.23 | 2.12 | 38.12 | 52.31 | 0.81 | 8.76 |
| Non-Muslim | 75.89 | 21.93 | 0.53 | 1.65 | 44.10 | 49.05 | 0.51 | 6.34 |
| **Disability status** |  |  |  |  |  |  |  |  |
| None | 74.00 | 22.44 | 1.22 | 2.35 | 44.17 | 47.62 | 0.76 | 7.44 |
| Some difficulty | 73.90 | 22.95 | 1.21 | 1.95 | 31.40 | 58.00 | 0.94 | 9.67 |
| Moderate/severe difficulty | 79.18 | 19.58 | 0.54 | 0.71 | 30.01 | 58.64 | 0.39 | 10.96 |
| **Husband’s education** |  |  |  |  |  |  |  |  |
| Primary or less | 69.92 | 26.56 | 1.14 | 2.38 | 32.24 | 57.63 | 0.67 | 9.46 |
| Secondary | 71.57 | 24.56 | 1.49 | 2.39 | 41.97 | 48.38 | 0.73 | 8.92 |
| Higher secondary+ | 77.34 | 19.43 | 1.36 | 1.86 | 52.17 | 40.97 | 1.18 | 5.69 |
| **Husband extra-marital relationship** |  |  |  |  |  |  |  |  |
| No | 76.70 | 20.33 | 1.18 | 1.80 | 41.92 | 49.83 | 0.84 | 7.42 |
| Yes | 50.38 | 43.84 | 0.83 | 4.95 | 5.47 | 74.53 | 0.15 | 19.85 |
| **Household type** |  |  |  |  |  |  |  |  |
| Non-slum | 74.46 | 22.32 | 1.16 | 2.07 | 38.96 | 51.74 | 0.79 | 8.51 |
| Slum | 74.61 | 22.57 | 0.80 | 2.02 | 32.94 | 60.05 | 0.13 | 6.88 |
| **Wealth quintile** |  |  |  |  |  |  |  |  |
| Poorest | 70.30 | 25.94 | 1.16 | 2.61 | 32.49 | 56.95 | 0.72 | 9.84 |
| Poorer | 74.32 | 22.11 | 1.33 | 2.25 | 36.82 | 53.76 | 1.14 | 8.28 |
| Middle | 75.65 | 21.43 | 1.29 | 1.63 | 40.58 | 50.99 | 0.49 | 7.94 |
| Richer | 76.99 | 20.55 | 0.95 | 1.52 | 43.08 | 48.46 | 0.83 | 7.63 |
| Richest | 76.91 | 20.05 | 0.85 | 2.20 | 45.71 | 45.33 | 0.50 | 8.46 |
| **Place of residence** |  |  |  |  |  |  |  |  |
| Rural | 74.58 | 22.20 | 1.23 | 1.99 | 38.84 | 51.79 | 0.83 | 8.55 |
| Urban | 74.17 | 22.62 | 0.97 | 2.24 | 38.78 | 52.26 | 0.66 | 8.30 |
| **Administrative division** |  |  |  |  |  |  |  |  |
| Barishal | 66.32 | 30.19 | 0.96 | 2.53 | 31.79 | 56.30 | 0.78 | 11.13 |
| Chattogram | 72.41 | 23.07 | 1.67 | 2.86 | 37.98 | 48.93 | 0.73 | 12.36 |
| Dhaka | 77.47 | 20.05 | 0.61 | 1.87 | 41.75 | 51.72 | 0.55 | 5.98 |
| Khulna | 73.12 | 23.04 | 1.53 | 2.30 | 32.81 | 55.24 | 0.66 | 11.30 |
| Mymensingh | 75.37 | 21.52 | 1.05 | 2.06 | 41.28 | 50.12 | 1.80 | 6.80 |
| Rajshahi | 78.47 | 18.97 | 1.37 | 1.18 | 38.01 | 55.52 | 0.98 | 5.49 |
| Rangpur | 70.44 | 26.48 | 1.10 | 1.98 | 38.57 | 52.28 | 0.63 | 8.51 |
| Sylhet | 74.18 | 22.88 | 1.37 | 1.58 | 46.39 | 43.57 | 0.83 | 9.20 |
