# Supplementary material for: Beyond conventional indicators: Reproductive coercion and its overlap with measured intimate partner violence in Bangladesh
Source: PLoS One. 2026 Oct 21;21(10):e0360372. doi: 10.1371/journal.pone.0360372 (PMC13686496; doi:10.1371/journal.pone.0360372)
Supplement: Supplementary file 18 [file pone.0360372.s018.docx]

Supplementary Table 18. Prevalence of reproductive coercion without reported conventional IPV by respondent characteristics among currently married women (past 12 months) and ever-married women (lifetime) in Bangladesh, 2024

| **Characteristics** | **Hidden RC in past 12 months % (95% CI)** | **Hidden lifetime RC % (95% CI)** |
| --- | --- | --- |
| **Women’s age (years)** |  |  |
| 15–19 | 3.02 (1.77–5.10) | 1.97 (0.93–4.14) |
| 20–29 | 1.59 (1.13–2.23) | 0.93 (0.63–1.37) |
| 30–39 | 1.55 (1.18–2.04) | 0.62 (0.40–0.95) |
| ≥40 | 0.44 (0.26–0.73) | 0.64 (0.41–0.98) |
| **Child marriage (marriage before 18 years)** |  |  |
| No | 1.22 (0.89–1.67) | 0.94 (0.64–1.37) |
| Yes | 1.12 (0.88–1.43) | 0.72 (0.54–0.97) |
| **Number of living children** |  |  |
| None | 1.16 (0.55–2.43) | 0.48 (0.17–1.39) |
| 1–2 | 1.14 (0.88–1.47) | 0.58 (0.42–0.80) |
| 3–4 | 1.12 (0.80–1.56) | 0.83 (0.55–1.26) |
| ≥5 | 0.04 (0.01–0.12) | 0.64 (0.34–1.20) |
| **Women’s education** |  |  |
| Primary or less | 0.72 (0.50–1.03) | 0.75 (0.52–1.09) |
| Secondary | 1.67 (1.30–2.13) | 0.80 (0.57–1.14) |
| Higher secondary+ | 1.34 (0.83–2.16) | 0.82 (0.51–1.33) |
| **Women’s employment status** |  |  |
| No | 1.17 (0.97–1.43) | 0.79 (0.61–1.03) |
| Yes | 0.59 (0.24–1.41) | 0.45 (0.16–1.22) |
| **Religion** |  |  |
| Muslim | 1.23 (1.01–1.50) | 0.81 (0.62–1.07) |
| Non-Muslim | 0.53 (0.30–0.95) | 0.51 (0.28–0.95) |
| **Disability status** |  |  |
| None | 1.22 (0.96–1.53) | 0.76 (0.53–1.07) |
| Some difficulty | 1.21 (0.86–1.70) | 0.94 (0.61–1.44) |
| Moderate/severe difficulty | 0.54 (0.29–0.99) | 0.39 (0.19–0.79) |
| **Husband’s education** |  |  |
| Primary or less | 1.14 (0.87–1.48) | 0.67 (0.46–0.96) |
| Secondary | 1.49 (1.10–2.00) | 0.73 (0.50–1.08) |
| Higher secondary+ | 1.36 (0.86–2.16) | 1.18 (0.60–2.32) |
| **Husband extra-marital relationship** |  |  |
| No | 1.18 (0.97–1.43) | 0.84 (0.64–1.09) |
| Yes | 0.83 (0.40–1.70) | 0.15 (0.04–0.63) |
| **Household type** |  |  |
| Non-slum | 1.16 (0.95–1.41) | 0.79 (0.61–1.03) |
| Slum | 0.80 (0.24–2.68) | 0.13 (0.02–0.96) |
| **Wealth quintile** |  |  |
| Poorest | 1.16 (0.80–1.67) | 0.72 (0.44–1.19) |
| Poorer | 1.33 (0.92–1.91) | 1.14 (0.69–1.87) |
| Middle | 1.29 (0.85–1.95) | 0.49 (0.27–0.90) |
| Richer | 0.95 (0.65–1.38) | 0.83 (0.55–1.25) |
| Richest | 0.85 (0.52–1.38) | 0.50 (0.31–0.82) |
| **Place of residence** |  |  |
| Rural | 1.23 (0.96–1.56) | 0.83 (0.60–1.14) |
| Urban | 0.97 (0.75–1.25) | 0.66 (0.47–0.95) |
| **Administrative division** |  |  |
| Barishal | 0.96 (0.59–1.55) | 0.78 (0.31–1.92) |
| Chattogram | 1.67 (1.18–2.34) | 0.73 (0.47–1.14) |
| Dhaka | 0.61 (0.41–0.90) | 0.55 (0.35–0.88) |
| Khulna | 1.53 (0.88–2.66) | 0.66 (0.33–1.30) |
| Mymensingh | 1.05 (0.41–2.66) | 1.80 (0.77–4.15) |
| Rajshahi | 1.37 (0.76–2.46) | 0.98 (0.46–2.08) |
| Rangpur | 1.10 (0.73–1.65) | 0.63 (0.37–1.06) |
| Sylhet | 1.37 (0.58–3.17) | 0.83 (0.21–3.20) |
