# Supplementary material for: Beyond conventional indicators: Reproductive coercion and its overlap with measured intimate partner violence in Bangladesh
Source: PLoS One. 2026 Oct 21;21(10):e0360372. doi: 10.1371/journal.pone.0360372 (PMC13686496; doi:10.1371/journal.pone.0360372)
Supplement: Supplementary file 19 [file pone.0360372.s019.docx]

**Supplementary Table 19. Factors associated with reproductive coercion without reported conventional intimate partner violence among currently married women (past 12 months) and ever-married women (lifetime) in Bangladesh, 2024: exploratory survey-weighted logistic regression models.**

| **Characteristic** | **Past 12 months, aOR (95% CI)** | **Lifetime, aOR (95% CI)** |
| --- | --- | --- |
| **Women’s age (years)** |  |  |
| 15–19 | Reference | Reference |
| 20–29 | 1.08 (0.48–2.44) | 5.17 (0.95–28.08)+ |
| 30–39 | 1.00 (0.43–2.35) | 3.03 (0.57–16.13) |
| ≥40 | 0.35 (0.12–1.04)+ | 2.17 (0.38–12.47) |
| **Child marriage (marriage before 18 years)** |  |  |
| No | Reference | Reference |
| Yes | 1.08 (0.71–1.64) | 0.63 (0.36–1.09)+ |
| **Number of living children** |  |  |
| None | Reference | Reference |
| 1–2 | 1.11 (0.47–2.63) | 1.23 (0.40–3.83) |
| 3–4 | 1.40 (0.54–3.58) | 1.67 (0.49–5.73) |
| ≥5 | 0.08 (0.02–0.40)** | 1.49 (0.34–6.62) |
| **Women’s education** |  |  |
| Primary or less | Reference | Reference |
| Secondary | 1.35 (0.78–2.33) | 0.57 (0.27–1.21) |
| Higher secondary+ | 1.66 (0.69–4.01) | 0.40 (0.12–1.34) |
| **Women’s employment status** |  |  |
| No | Reference | Reference |
| Yes | 0.83 (0.35–1.97) | 1.03 (0.34–3.13) |
| **Religion** |  |  |
| Muslim | Reference | Reference |
| Non-Muslim | 0.55 (0.30–1.03)+ | 0.70 (0.32–1.53) |
| **Disability status** |  |  |
| None | Reference | Reference |
| Some difficulty | 1.84 (1.26–2.70)** | 1.57 (0.91–2.73) |
| Moderate/severe difficulty | 1.57 (0.75–3.27) | 0.36 (0.13–1.01)+ |
| **Husband’s education** |  |  |
| Primary or less | Reference | Reference |
| Secondary | 1.00 (0.61–1.62) | 0.98 (0.51–1.88) |
| Higher secondary+ | 0.69 (0.36–1.33) | 1.62 (0.58–4.51) |
| **Husband extra-marital relationship** |  |  |
| No | Reference | Reference |
| Yes | 0.71 (0.36–1.39) | 0.01 (0.001–0.04)*** |
| **Household type** |  |  |
| Non-slum | Reference | Reference |
| Slum | 1.29 (0.56–2.95) | 0.34 (0.04–2.55) |
| **Wealth quintile** |  |  |
| Poorest | Reference | Reference |
| Poorer | 0.98 (0.58–1.67) | 2.05 (0.92–4.56)+ |
| Middle | 1.02 (0.54–1.93) | 0.86 (0.34–2.17) |
| Richer | 0.68 (0.36–1.30) | 1.82 (0.78–4.26) |
| Richest | 0.68 (0.31–1.48) | 0.95 (0.33–2.78) |
| **Place of residence** |  |  |
| Rural | Reference | Reference |
| Urban | 0.89 (0.61–1.30) | 1.15 (0.70–1.88) |
| **Administrative division** |  |  |
| Barishal | Reference | Reference |
| Chattogram | 2.09 (1.07–4.07)* | 0.85 (0.25–2.88) |
| Dhaka | 0.58 (0.29–1.17) | 0.57 (0.17–1.95) |
| Khulna | 1.92 (0.83–4.42) | 0.91 (0.22–3.69) |
| Mymensingh | 0.71 (0.23–2.24) | 1.79 (0.49–6.58) |
| Rajshahi | 1.41 (0.57–3.54) | 0.67 (0.16–2.89) |
| Rangpur | 1.47 (0.74–2.91) | 0.98 (0.29–3.34) |
| Sylhet | 1.78 (0.62–5.13) | 1.13 (0.20–6.46) |

**Abbreviation:** aOR, adjusted odds ratio.

**Note:**  Estimates were obtained from survey-weighted multivariable logistic regression models adjusted for all characteristics shown, accounting for sampling weights, stratification, and clustering. The unweighted numbers of events and non-events were examined before interpretation, both overall and within each covariate category, and model estimates were checked against these counts
